# L‐serine biosynthesis in the human central nervous system: Structure and function of phosphoserine aminotransferase

**DOI:** 10.1002/pro.4609

**Published:** 2023-04-01

**Authors:** Francesco Marchesani, Erika Zangelmi, Giulia Murtas, Elisa Costanzi, Raheem Ullah, Alessio Peracchi, Stefano Bruno, Loredano Pollegioni, Andrea Mozzarelli, Paola Storici, Barbara Campanini

**Affiliations:** ^1^ Department of Food and Drug University of Parma Parma Italy; ^2^ Department of Chemistry, Life Sciences and Environmental Sustainability University of Parma Parma Italy; ^3^ Department of Biotechnology and Life Sciences University of Insubria Varese Italy; ^4^ Protein Facility, Elettra ‐ Sincrotrone Trieste S.C.p.A Trieste Italy; ^5^ Institute of Biophysics, CNR Pisa Italy; ^6^ Present address: Structural Biology Lab, NIBGE Faisalabad Pakistan

**Keywords:** neurological disorders, NMDA receptor, phosphorylated pathway, phosphoserine aminotransferase, tumor progression factor

## Abstract

Organisms from all kingdoms of life synthesize L‐serine (L‐Ser) from 3‐phosphoglycerate through the phosphorylated pathway, a three‐step diversion of glycolysis. Phosphoserine aminotransferase (PSAT) catalyzes the intermediate step, the pyridoxal 5′‐phosphate‐dependent transamination of 3‐phosphohydroxypyruvate and L‐glutamate to *O*‐phosphoserine (OPS) and α‐ketoglutarate. PSAT is particularly relevant in the central nervous system of mammals because L‐Ser is the metabolic precursor of D‐serine, cysteine, phospholipids, and nucleotides. Several mutations in the human *psat* gene have been linked to serine deficiency disorders, characterized by severe neurological symptoms. Furthermore, PSAT is overexpressed in many tumors and this overexpression has been associated with poor clinical outcomes. Here, we report the detailed functional and structural characterization of the recombinant human PSAT. The reaction catalyzed by PSAT is reversible, with an equilibrium constant of about 10, and the enzyme is very efficient, with a *k*_cat_/*K*_m_ of 5.9 × 10^6^ M^−1^ s^−1^, thus contributing in driving the pathway towards the products despite the extremely unfavorable first step catalyzed by 3‐phosphoglycerate dehydrogenase. The 3D X‐ray crystal structure of PSAT was solved in the substrate‐free as well as in the OPS‐bound forms. Both structures contain eight protein molecules in the asymmetric unit, arranged in four dimers, with a bound cofactor in each subunit. In the substrate‐free form, the active site of PSAT contains a sulfate ion that, in the substrate‐bound form, is replaced by the phosphate group of OPS. Interestingly, fast crystal soaking used to produce the substrate‐bound form allowed the trapping of different intermediates along the catalytic cycle.

## Abstract 2

PDB Code(s): 8A5V; 8A4W

## 1 INTRODUCTION

L‐serine (L‐Ser) is produced through different biosynthetic routes, including the widespread three‐step phosphorylated pathway (PP), which uses the glycolytic intermediate 3‐phosphoglycerate (3‐PG) as the precursor. In the mammalian central nervous system (CNS), the PP is crucial for maintaining the levels of L‐Ser, as this nonessential amino acid is transported inefficiently through the blood–brain barrier. The first step of the PP is catalyzed by 3‐PG dehydrogenase (PHGDH; Murtas et al., 2021; Unterlass et al., 2017) which oxidizes 3‐PG to 3‐phosphohydroxypyruvate (3‐PHP) using NAD^+^ as the electron acceptor. The equilibrium of this reaction is strongly shifted in favor of 3‐PG (Grant, 2012; Murtas et al., 2020); therefore, the subsequent reactions of the pathway must provide a thermodynamic drive to synthesize L‐Ser. Indeed, the second reaction, catalyzed by phosphoserine aminotransferase (PSAT), shows an equilibrium shifted towards the products L‐*O‐*phosphoserine (OPS) and α‐ketoglutarate (α‐KG; Merrill et al., 1981). The final step of the PP is the hydrolysis of the phosphate group of OPS by phosphoserine phosphatase (Marchesani et al., 2021) with formation of L‐Ser. In the human CNS, the PP takes place in the astrocytes and the L‐Ser thus produced is used locally or transported to neurons, where it can be converted to its enantiomer D‐Ser by serine racemase. (Marchesani, Gianquinto, et al., 2021; Raboni et al., 2018)

Human PSAT (EC 2.6.1.52) is a pyridoxal 5′‐phosphate (PLP)‐dependent enzyme whose coding gene is located on the long arm of chromosome 9. The primary gene transcript, and probably the only physiologically relevant one (Baek et al., 2003), codes for PSAT1 (or PSAT‐β). Mutations in the *psat* gene have been identified in neurological diseases characterized by low L‐Ser and glycine (Gly) levels in the cerebrospinal fluid and in the plasma. Most of the mutations are incompatible with life, and even the milder ones still cause very severe symptoms (Abdelfattah et al., 2020; Acuna‐Hidalgo et al., 2014; Brassier et al., 2016; Debs et al., 2021; Glinton et al., 2018; Hart et al., 2007; Ni et al., 2019; Shapira Zaltsberg et al., 2020; Sirr et al., 2020). On the other hand, the increased PSAT activity in certain cancer types has been linked to tumor progression, poor prognosis, and poor response to therapy (de Marchi et al., 2017; Liu et al., 2016; Martens et al., 2005; Ravez et al., 2017; Rossi et al., 2022; Tajan et al., 2021). Despite this, no selective PSAT inhibitor has been identified to date.

**SCHEME 1:**
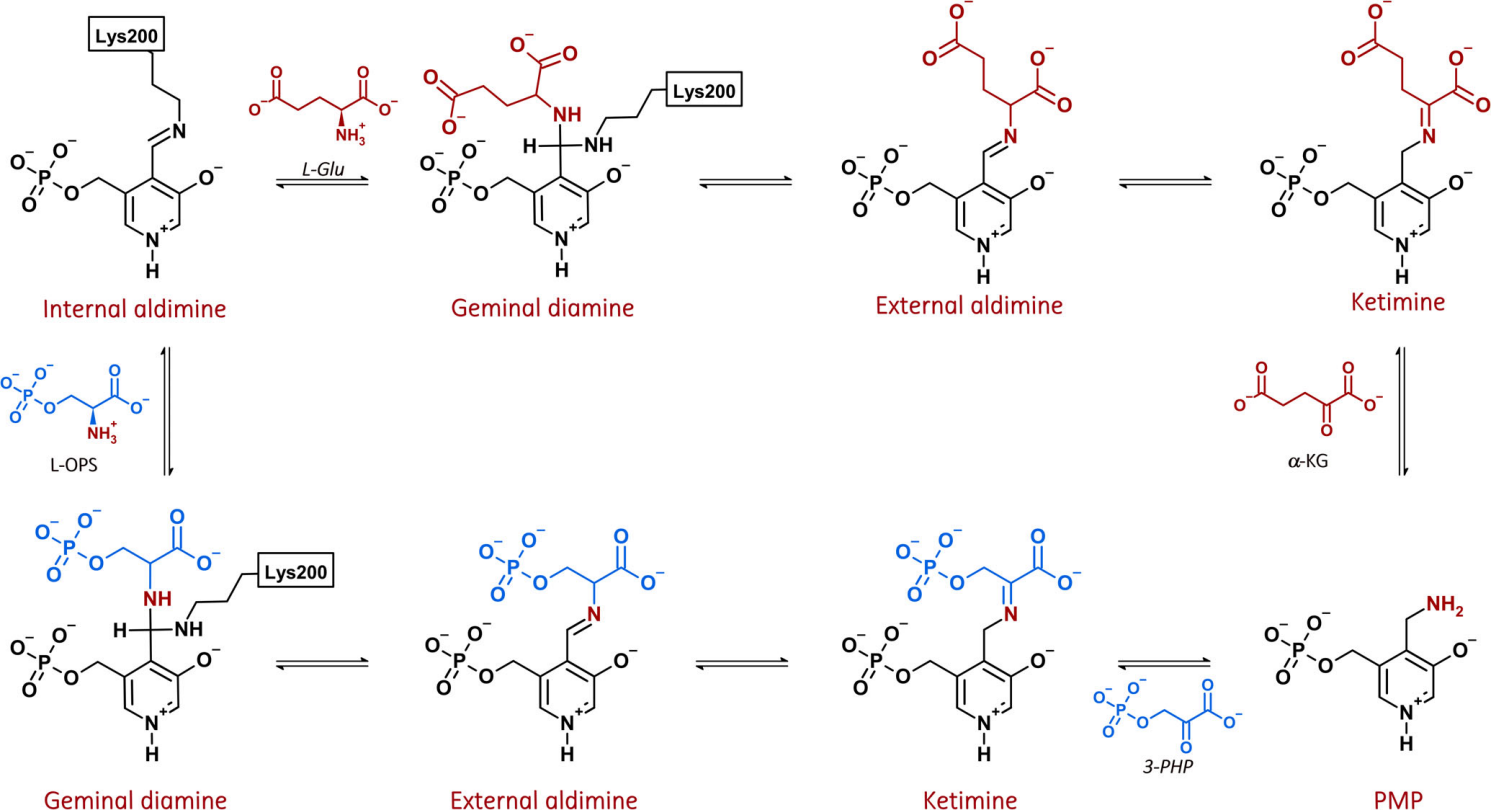
Proposed mechanism of the reaction catalyzed by phosphoserine aminotransferase. 3‐PHP, 3‐phosphohydroxypyruvate; L‐Glu, L‐glutamate; L‐OPS, L‐*O*‐phosphoserine; PMP, pyridoxamine phosphate.

The catalytic mechanism and the functional parameters were defined for several PSATs from eukaryotes and bacteria (Ali & Nozaki, 2006; Basurko et al., 1989; Basurko et al., 1999; Fell & Snell, 1988; Haque et al., 2019; Hirsch & Greenberg, 1967; Koivulehto et al., 2020; Singh et al., 2019; Snell & Fell, 1990), but this information is lacking for the human enzyme (Baek et al., 2003; Donini et al., 2009). In analogy to aspartate transaminase and other PLP‐dependent aminotransferases, PSAT follows a ping–pong mechanism, with L‐glutamate (L‐Glu) binding first (Basurko et al., 1989). This half‐reaction leads to the formation of pyridoxamine phosphate (PMP), which subsequently reacts with the second substrate, 3‐PHP, to form OPS and regenerate the PLP internal aldimine (Scheme 1).

The only complete characterization of a mammalian PSAT available to date was published by Basurko and collaborators on the enzyme purified from bovine liver (Basurko et al., 1989; Basurko et al., 1999). Furthermore, very little is known about the enzyme regulation by physiologically relevant metabolites, with many studies focusing on the reactivity towards amino acid substrates. In this respect, human PSAT is quite specific for 3‐PHP and L‐Glu as substrates, with moderate activity on L‐aspartate, L‐alanine, and L‐Ser (Caligiore et al., 2022; Donini et al., 2009).

While the Protein Data Bank (PDB) contains several crystal structures of PSAT enzymes from unicellular organisms (including *Escherichia coli*, *Mycobacterium tuberculosis*, *Bacillus alcalophilus*, and *Entamoeba histolytica*; Battula et al., 2013; Coulibaly et al., 2012; Dubnovitsky et al., 2003; Dubnovitsky, Kapetaniou, & Papageorgiou, 2005; Dubnovitsky et al., 2005; Hester et al., 1999; Kapetaniou et al., 2006; Singh et al., 2019) and plants (such as *Arabidopsis thaliana*; Sekula et al., 2018), the only available mammalian structure is the human ortholog (PDB ID: 3E77), deposited by the Structural Genomic Consortium with no associated publication. All the orthologs are consistently dimeric, with the cofactor bound at the monomer–monomer interface forming polar contacts with residues from both chains. The active site entrance is positively charged to favor the entry of the negatively charged substrates (Coulibaly et al., 2012; Sekula et al., 2018). The enzyme binds its phosphorylated substrates/products (3‐PHP and OPS) with significantly higher affinity as compared with the bi‐carboxylic ones (L‐Glu and α‐KG). Indeed, in the *B. alcalophilus* enzymes a set of conserved residues (that correspond to Arg45, Arg336, His44, and His335 in the human sequence) provides a strong anchoring to the active site for the phosphate group of the substrate (Battula et al., 2013). In the deposited human PSAT structure solved at 2.5 Å in the substrate‐free, PLP‐form, the side chains of Arg45 and Arg336 could not be modeled, thus hampering a full elucidation of the substrate‐binding mode and interactions. Furthermore, this structure contains a cloning‐derived N‐terminal sequence that influences its conformation. As a result, the available model of human PSAT is not suitable for the in silico identification of enzyme inhibitors. In this work, we report a detailed functional and spectroscopic characterization of recombinant human PSAT and two new crystal structures of the wild‐type enzyme in the substrate‐free form and in complex with OPS.

## 2 RESULTS

### 2.1 Substrate binding, thermal stability, and spectroscopic features of human PSAT

**FIGURE 1:**
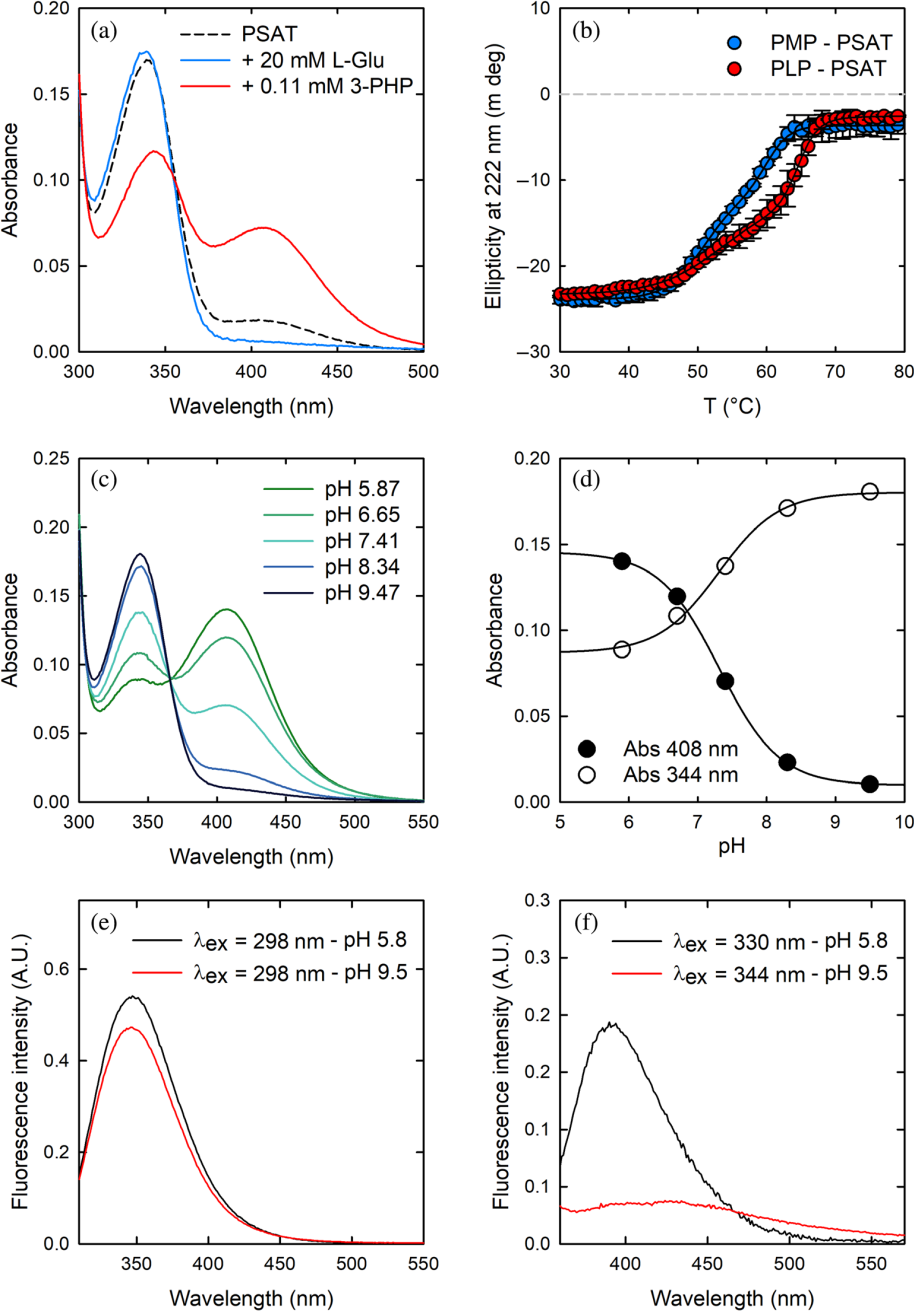
Spectroscopic characterization of human phosphoserine aminotransferase (PSAT). (a) Absorption spectra of 40 μM PSAT in 20 mM potassium phosphate pH 7.0, in the absence (dashed black line) and presence of either 20 mM L‐glutamate (L‐Glu; blue line) or 0.11 mM 3‐phosphohydroxypyruvate (3‐PHP; red line). (b) Thermal unfolding of pyridoxamine phosphate (PMP)‐PSAT (blue circles) and pyridoxal 5′‐phosphate (PLP)‐PSAT (red circles) monitored by circular dichroism at 222 nm. The continuous lines are the fitting of data points to Equation (2) with the following melting temperatures: 50.9 ± 0.7°C and 60.1 ± 0.5°C for PMP‐PSAT, 53.4 ± 1.7°C and 64.6 ± 0.3°C for PLP‐PSAT. (c) Absorption spectra of 47 μM PLP‐PSAT at different pH values. (d) pH‐dependence of the absorption at 344 nm (open circles) and 408 nm (closed circles). The solid lines are the fitting of the data points to Equation (1) with p*K*_a_ 7.30 ± 0.07 (344 nm) and 7.31 ± 0.01 (408 nm). (e) Fluorescence emission spectra of 30 μM PSAT excited at 298 nm at pH 5.8 (black line) and 9.5 (red line). (f) Fluorescence emission spectra of 30 μM PSAT excited at 330 nm (slits = 5) at pH 5.8 (black line) and at 344 nm (slits = 5) at pH 9.5 (red line).

Human PSAT shows the typical absorption spectrum of transaminases, with two peaks in the visible region centered at ~339 and 408 nm (Figure 1a).

The peak at 408 nm is observed in most PLP‐dependent enzymes and is attributed to the cofactor bound as internal aldimine to the catalytic lysine (Lys200) in a protonated, ketoenamine form (Scheme S1). At least three forms of the cofactor might contribute to the absorption in the spectral region between 330 and 345 nm: the enolimine tautomer of the protonated internal aldimine of PLP (λ_max_ = 330 nm), the deprotonated internal aldimine (λ_max_ = 345 nm) and the PMP form (λ_max_ = 340 nm; Dubnovitsky et al., 2005; Jenkins & Sizer, 1959, 1960). The peak at 339 nm was more intense for the protein purified in Tris buffer, suggesting the presence of the PMP‐enzyme form under these conditions. The near‐UV circular dichroism spectrum of PLP‐PSAT showed two positive dichroic peaks at ~339 and 408 nm (Figure S1). The addition of 0.11 mM 3‐PHP to the solution containing 40 μM PSAT led to a pronounced red‐shift of the band at 339 nm to 344 nm and to an increase in the intensity of the band at 408 nm, indicating a conversion of PMP to the PLP form (Figure 1a). On the other hand, the addition of 20 mM L‐Glu led to the disappearance of the band at 408 nm and to a small increase in the intensity of the band at 339 nm, indicative of the conversion of PLP to the PMP form (Figure 1a).

The far‐UV circular dichroism spectra of PLP‐PSAT and PMP‐PSAT, prepared by treating with either 3‐PHP or L‐Glu, were almost superimposable, indicating a negligible effect of the cofactor form on the secondary structure content of the folded protein (Figure S1). Thermal denaturation of the two protein forms was monitored by circular dichroism spectroscopy at 222 nm (Figure 1b). Both showed a three‐state melting curve, with formation of an intermediate that was more stable in the case of PLP‐PSAT. Indeed, the first phase for both PMP‐bound and PLP‐bound PSAT was almost superimposable, whereas the *T*_m_ of the second phase was about 4°C higher for the PLP‐form. This behavior suggests that the covalently bound PLP may stabilize, more than PMP, a partially unfolded dimeric intermediate, as shown for the *E. histolytica* enzyme (Mishra et al., 2011).

The absorption spectrum of PLP‐PSAT at pH 7.0 showed contributions from both the protonated and deprotonated forms of the PLP‐internal aldimine (Figure 1c and Scheme S1), an indication that the p*K*_a_ for this equilibrium is significantly lower than the average p*K*_a_ for an imine, which is around 10–12 (Hayashi et al., 1998, 2003). Lowering the pH from 9.5 to 5.8 caused a decrease of the peak centered at 344 nm and a concomitant increase of the peak at 408 nm, with an isosbestic point at 365 nm, indicating an equilibrium between only two chemical species. The p*K*_a_ for this protonation equilibrium was 7.3 ± 0.1 (Figure 1d). At pH 5.9, a shoulder centered at about 340 nm remained visible, suggesting that a fraction of the cofactor might still be in the PMP form. The presence of a fraction of cofactor in the PMP form was also apparent in the crystal structure, where about 25% of active sites contained PMP (vide infra).

The human PSAT sequence possesses three tryptophan residues (Trp107, Trp140, and Trp257), all located in the large, N‐terminal domain. Trp107 is in the active site, with its indole ring stacking against the pyridine ring of the cofactor (vide infra), and plays an important role in cofactor orientation and catalysis (Mishra et al., 2012). We collected fluorescence excitation and emission spectra of PSAT (Figures 1e and S2). The emission spectrum of Trp residues upon excitation at 298 nm showed a peak centered at 346 nm, irrespective of pH, that corresponds to the direct emission of Trp residues, with no evidence of energy transfer to the cofactor (Mishra et al., 2010). At pH 5.8, PLP emitted fluorescence with a maximum at 392 nm only when excited at 330 nm, with no appreciable emission when excited at 408 nm. This observation suggests that the predominant form of PLP in the excited state is the enolimine, rather than the ketoenamine. At pH 9.5, PLP showed a maximum of excitation at 344 nm, consistent with the absorption spectrum recorded at the same pH, which was associated with a very modest emission at around 425 nm (Figures 1f and S2).

### 2.2 Human PSAT efficiently catalyzes the transamination of 3‐PHP using L‐Glu as amino donor

**FIGURE 2:**
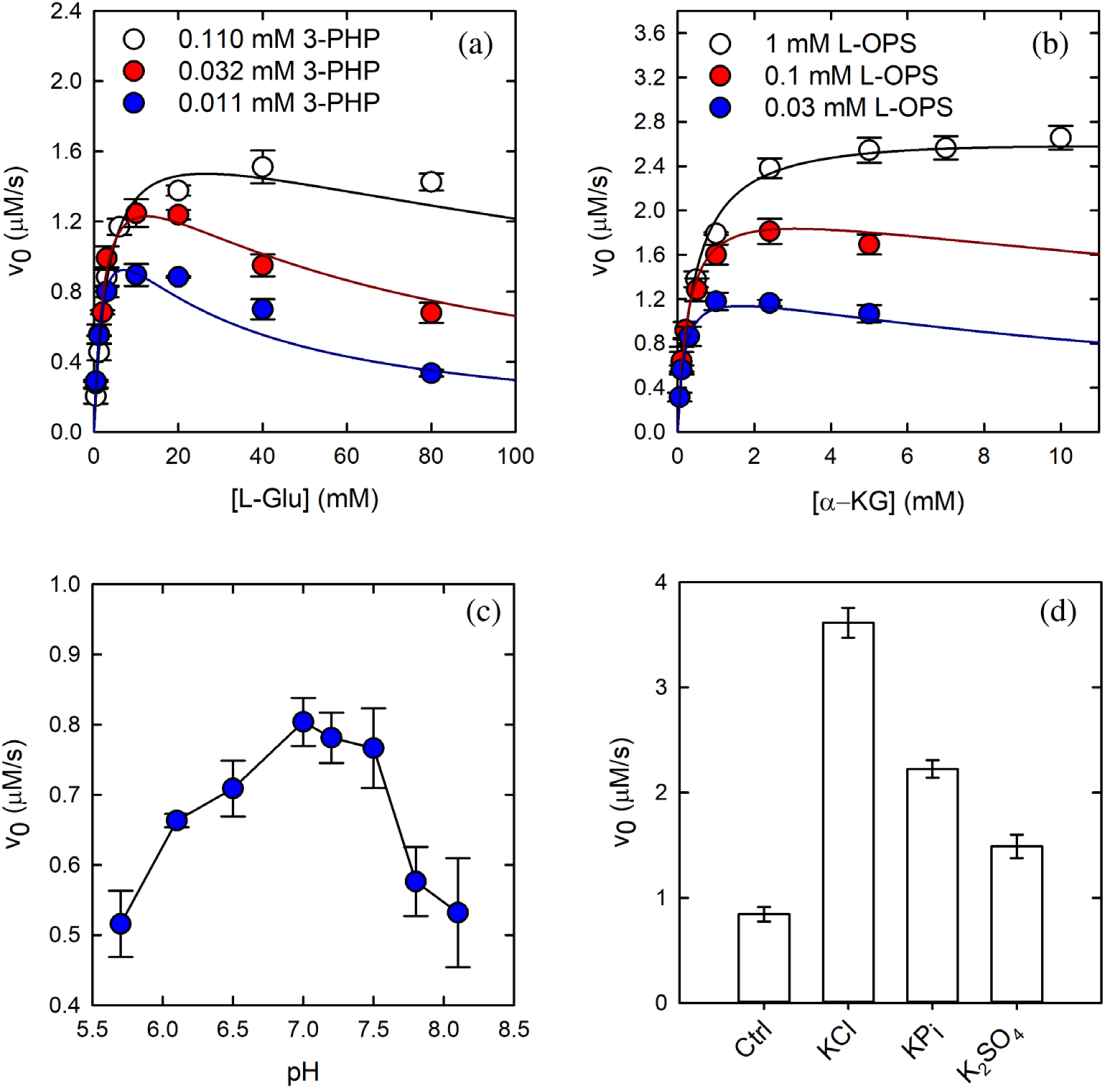
Functional characterization of human phosphoserine aminotransferase (PSAT). (a) Dependence of the initial velocity of the forward reaction catalyzed by PSAT on L‐glutamate (L‐Glu) concentration in the presence of 0.110 mM (open circles), 0.032 mM (red circles), and 0.011 mM (blue circles) 3‐phosphohydroxypyruvate (3‐PHP). (b) Dependence of the initial velocity of the reverse reaction catalyzed by PSAT on α‐ketoglutarate (α‐KG) concentration in the presence of 1 mM (open circles), 0.1 mM (red circles), and 0.03 mM (blue circles) L‐*O*‐phosphoserine (L‐OPS). The solid lines in (a) and (b) are the fitting of data points to Equation (3). (c) Dependence of the initial velocity of PSAT (forward reaction) on pH, in the presence of 20 mM L‐Glu and 0.11 mM 3‐PHP. (d) Effect of either 0.2 M potassium phosphate or 0.2 M potassium sulfate on the activity of 336 nM PSAT (reverse reaction) in the presence of 1 mM L‐OPS and 5 mM α‐KG. The activity in the absence of added salts (ctrl) and in the presence of 0.2 M KCl is reported for comparison.

PSAT catalyzes a reversible reaction using 3‐PHP/Glu and α‐KG/OPS in the forward and reverse directions, respectively (Scheme 1 and Figure S3). Our preliminary results indicated that α‐KG at concentrations above 4 mM inhibits the reaction. To account for this effect and detect any substrate inhibition that might be physiologically relevant, we determined the intrinsic kinetic and substrate inhibition constants by varying the concentration of both substrates simultaneously and by globally fitting the dependences of the observed rate on substrate concentration to the equation for a ping–pong mechanism with substrate inhibition (Equation 3). The results for both the forward and the reverse reactions are shown in Figure 2a,b and the kinetic parameters are reported in Table 1. Substrate inhibition was observed for both substrates in the forward and reverse directions. The very low *K*_m,3‐PHP_ affected the accuracy of the determination, as mirrored by the very high standard error (Table 1). We assessed the *K*_m,3‐PHP_ and *k*_cat_ values through an independent method, that is, by fitting the time course of α‐KG accumulation to the integrated Michaelis–Menten equation (Goličnik, 2013; Schnell & Mendoza, 1997; Figure S3c).

**TABLE 1:** Kinetic and inhibition constants for the forward and reverse reactions catalyzed by PSAT at 37°C in a solution containing 50 mM HEPES (4‐(2‐hydroxyethyl)piperazine‐1‐ethanesulfonic acid), 100 mM KCl, 1 mM DTT (dithiothreitol), 0.17 mM PLP, and pH 7.0.

Substrate	*k*_cat_/*K*_m_ (M^−1^ s^−1^)	*K*_m_ (mM)	*K*_i_ (mM)	Physiological concentration (mM)^a^
Forward reaction				
L‐Glu	9.9 × 10^3^	2.4 ± 0.4	7.1 ± 3.9	1.5^†^
3‐PHP	5.9 × 10^6^	0.004 ± 0.002	0.23 ± 0.14	0.00025^‡^
Reverse reaction				
OPS	2.6 × 10^5^	0.033 ± 0.005	3.4 ± 2.0	0.005^§^
α‐KG	2.4 × 10^4^	0.36 ± 0.04	8.4 ± 4.7	0.32^†^

*Note*: The calculated *k*_cat_ values, not reported in the table, were 23.8 ± 1.2 s^−1^ for the forward reaction and 8.6 ± 0.2 s^−1^ for the reverse reaction. Physiological substrate concentrations of the four substrates, determined in other studies, are shown for convenience in the last column.

Abbreviations: 3‐PHP, 3‐phosphohydroxypyruvate; L‐Glu, L‐glutamate; OPS, *O‐*phosphoserine; PLP, pyridoxal 5′‐phosphate; PSAT, phosphoserine aminotransferase; α‐KG, α‐ketoglutarate.

^a^ Concentrations refer to ^†^murine hybridoma (Zupke et al., 1995); ^‡^rat brain (Merrill et al., 1981); ^§^human cerebrospinal fluid (Wishart et al., 2022).

The parameters calculated by this method (*K*_m,3‐PHP_ = 6.9 ± 0.5 μM and *k*_cat_ = 19 ± 0.7 s^−1^) were in good agreement with those obtained from global fitting. The equilibrium constant of the reaction calculated at 37°C, pH 7 using the Haldane equation for a ping–pong mechanism (Equation 5) was 9.6 ± 5.4, in very good agreement with the *K*_eq_ calculated from the concentrations of 3‐PHP and α‐KG at equilibrium, that is, 11.3 ± 0.3 (Table S1 and Figure S4). The consistency between the values calculated by independent methods supports the validity of the kinetic model. The dependence of the forward reaction on pH is bell‐shaped, suggesting that the activity is apparently controlled by two ionizable groups of the substrate–enzyme complex (Figure 2c). However, the small amplitude of the activity change as a function of pH does not allow to calculate accurate p*K*_a_ values. Maximum activity occurred at pH 6.9, where about 70% of internal aldimine is in the protonated state (Figure 1d), similarly to what is observed for many aminotransferases, except aspartate aminotransferase (Hester et al., 1999).

### 2.3 Alternative substrates and activity modulators

The reactivity of PSAT with different substrates and the identification of biologically relevant modulators of activity have been poorly investigated. Donini et al. (2009) examined the ability of PSAT to transaminate the 20 proteinogenic amino acids using glyoxylate as the amino group acceptor. In addition to L‐Glu, PSAT transaminated L‐aspartate, L‐alanine, and L‐Ser. Cysteine reacted with PSAT to form the PMP intermediate (Figure S5a) and did not lead to cofactor release by formation of the thiazolidine ring typical of many PLP‐dependent enzymes (Lowther et al., 2012). Cysteine sulfinate (CSA), a *N*‐methyl‐D‐aspartate agonist and a compound involved in taurine biosynthesis, was also reported to react with the enzyme, and indeed it has been used to prepare the apoenzyme form (Deu & Kirsch, 2007; Mishra et al., 2011). We found that CSA reacts with PSAT to form the PMP intermediate, but the transamination reaction with 3‐PHP is extremely inefficient, with a *k*_cat_/*K*_m_ of 58.1 M^−1^ s^−1^ (Figure S5b).

Considering that bovine PSAT binds Cibacron blue‐agarose (Basurko et al., 1999; Lund et al., 1987), a dye commonly used to purify nucleotide‐binding proteins (Thresher & Swaisgood, 1983), we investigated the effect of selected nucleotides on the activity of PSAT. We found that neither NAD^+^/NADH, nor ATP/AMP exerted any effect on the activity of PSAT (see Section S3.1 and Figure S6a). On the other hand, we observed that salts in general, and halides in particular, increased the activity of PSAT, likely as result of the effect of the ionic strength (see Section S3.1 and Figure S6b–d).

**FIGURE 3:**
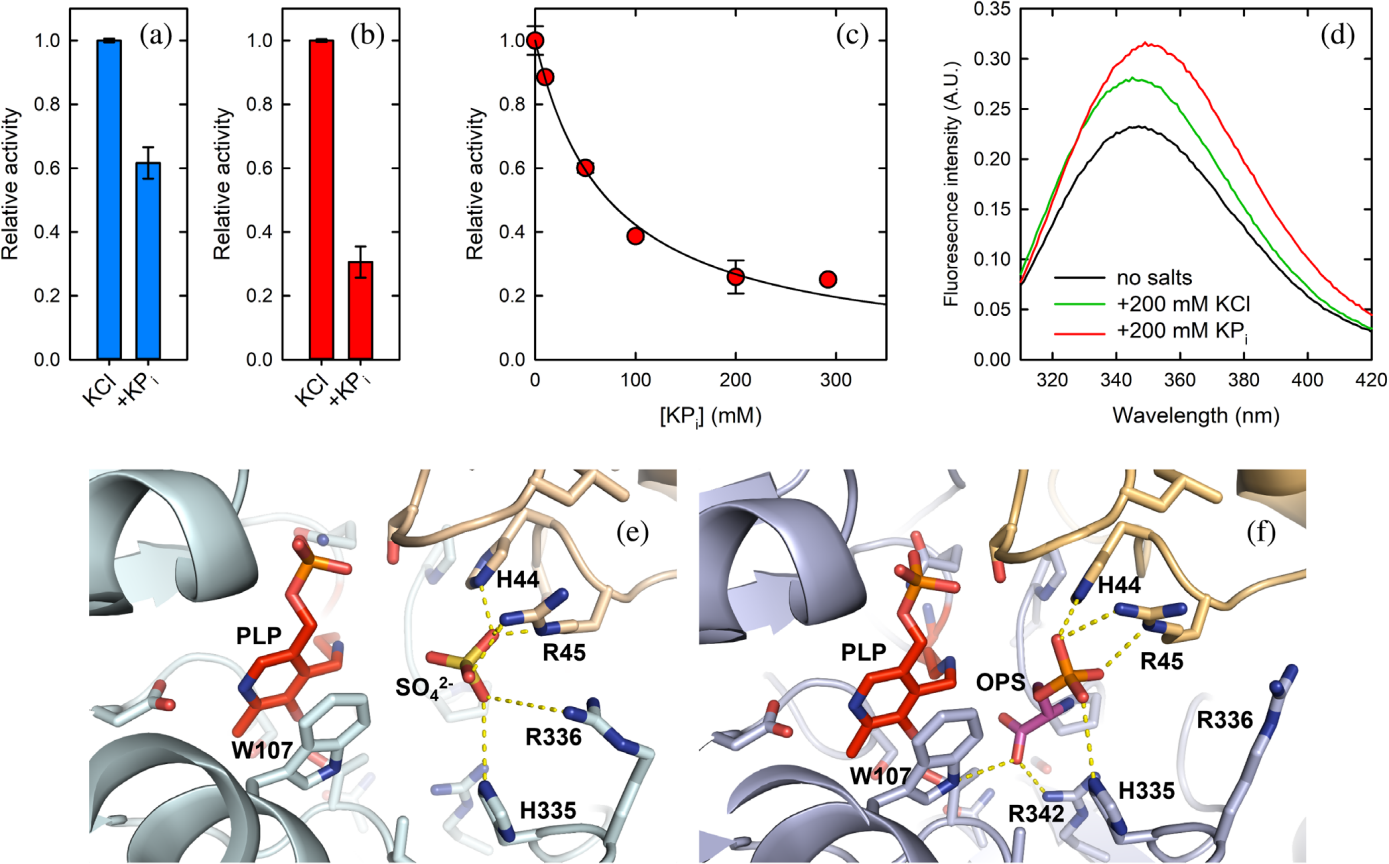
Effect of phosphate on phosphoserine aminotransferase (PSAT) activity. (a) Activity of PSAT at saturating substrate concentrations (reverse reaction) in the presence of either 0.2 M KCl alone or added of 0.2 M KP_i_. (b) Activity of PSAT at substrate concentrations equal to the *K*_m_ (reverse reaction) in the presence of either 0.2 M KCl alone or added of 0.2 M KP_i_. (c) Dependence of the initial velocity of the reverse reaction catalyzed by PSAT on potassium phosphate concentration. Substrate concentrations were equal to the *K*_m_ for both L‐*O*‐phosphoserine (L‐OPS) and α‐ketoglutarate. Line through data points is the fitting to equation 6 with IC_50_ = 73 ± 5 mM. (d) Fluorescence emission spectra of PSAT (5 μM) in the absence of added salts (black line), in the presence of KCl (green line) or in the presence of KP_i_ (red line). (e) Close‐up of the active site of PSAT bound to a sulfate ion (SO_4_). (f) Close‐up of the active site of PSAT bound to L‐OPS. PLP, pyridoxal 5′‐phosphate.

In the X‐ray crystal structure of human PSAT (see below), a sulfate ion from the crystallization solution was bound at the active site near the cofactor, interacting with some of the residues that are part of the phosphate binding site in the OPS‐bound structure (vide infra). We thus tested the effect on the reverse reaction of sulfate and phosphate, at the same concentration used in the crystallization solution (i.e., 200 mM). In the absence of any added salts, phosphate and sulfate both activated the enzyme by about 2‐fold and 1.5‐fold, respectively. For comparison, the effect brought about by 200 mM KCl is a 4‐fold to 5‐fold activation (Figure 2d). Considering KCl as reference, sulfate and phosphate both inhibited the activity of the enzyme to a similar extent. This inhibition was competitive with respect to the phosphorylated substrate (i.e., OPS). As a matter of fact, the inhibitory effect of phosphate was larger at lower substrate concentrations (Figure 3a,b). The IC_50_ for phosphate, measured in the presence of 200 mM KCl to saturate any nonspecific ionic strength effects, was 73 ± 5 mM, (Figure 3c). To assess the effect of phosphate on the enzyme conformation, we collected fluorescence emission spectra upon excitation at 298 nm in the presence of either KCl or potassium phosphate (Figure 3d). The addition of 200 mM potassium phosphate caused an increase in the emission maximum and a 5‐nm red shift of the spectrum (Figure 3d). Conversely, the addition of 200 mM KCl caused a less pronounced increase in the emission intensity, with no shift in the emission maximum.

### 2.4 Overall structure of substrate‐free and OPS‐bound PSAT

**FIGURE 4:**
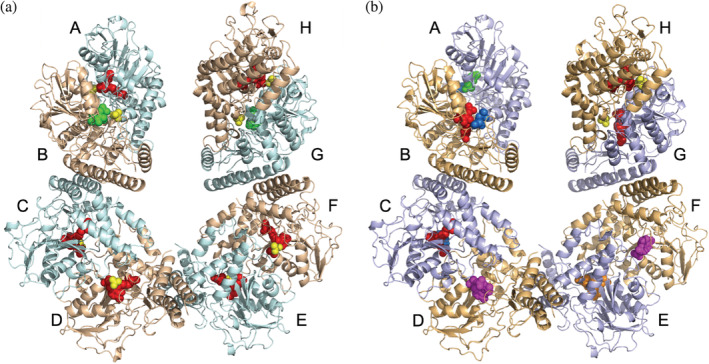
Cartoon model of the four dimers in the asymmetric unit. (a) Unbound phosphoserine aminotransferase (PSAT). (b) *O*‐phosphoserine (OPS)‐bound PSAT, with OPS shown in blue marine, external aldimine shown in magenta, geminal diamine shown in orange. In both panels pyridoxal 5′‐phosphate‐ Lys200 is shown in red, pyridoxamine phosphate is shown in green, and sulfate is shown in yellow.

The PSAT crystals, obtained in space group P2_1_, contained four dimers (eight protomers) in the asymmetric unit (Figure 4). All monomers appeared well ordered, showing no interruptions in the electron density of the main chains from residue 6 to 370. Each monomer exhibits the well‐conserved α/β fold of fold‐type I PLP‐enzymes with two distinct domains. The large domain (residues 19–265) is composed of seven‐stranded β sheets flanked by six α‐helices, which mainly contribute to the dimer interface. The small domain, formed by the C‐terminal region (266–307) plus the first eighteen N‐terminal residues, folds into three antiparallel β‐strands flanked by three α‐helices. The eight monomers are organized in four S‐shaped dimers, each constituting the stable assembly in solution, as suggested by the PISA server (Krissinel, 2015) and confirmed by size‐exclusion chromatography (Figure S7). The four dimers, as well as the eight monomers, were virtually identical, with root mean square deviation (RMSD) ranging from 0.142 to 0.210 Å (Tables S2 and S4).

**FIGURE 5:**
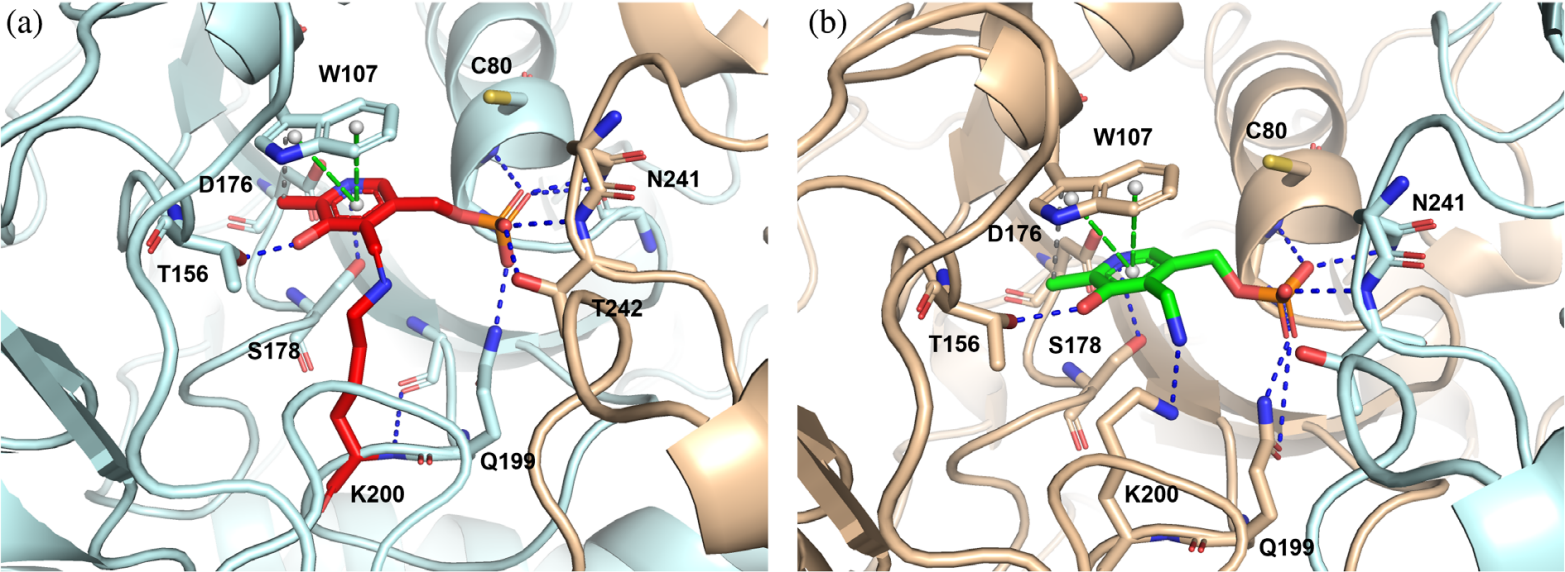
Close‐up of the active site of the substrate‐free phosphoserine aminotransferase. (a) Subunit A with pyridoxal 5′‐phosphate‐ Lys200 (red) in the active site (b) Subunit B with pyridoxamine phosphate (green) in the active site. The interactions of the cofactor with active site residues (in ball and stick) are shown as dotted lines.

In the substrate‐free structure, solved at 2.5 Å, the main differences between the eight chains were found in the catalytic site and involved the state of the cofactor: in chains A, C, D, E, F, and H, the electron density clearly showed PLP covalently bound to Lys200, forming the internal aldimine. Instead, in chains B and G, PMP was present in place of PLP (Figures 4 and 5). These results agree with the spectroscopic analysis indicating the co‐presence of PLP and PMP.

The crystal structure of PSAT in the OPS‐bound form was obtained by briefly soaking native crystals in 50 mM OPS. Longer soakings made the crystals diffract poorly or completely dissolve. The best crystal diffracted to 2.78 Å resolution, preserving the same cell parameters and space group with eight chains per asymmetric unit. As for the substrate‐free form, all chains superimpose well, with minimal RMSD variations (Tables S3 and S5). Due to the very short soaking time, we could capture subsequent steps of the OPS reaction (Figure 4), observing differences between the catalytic sites of the eight chains.

**FIGURE 6:**
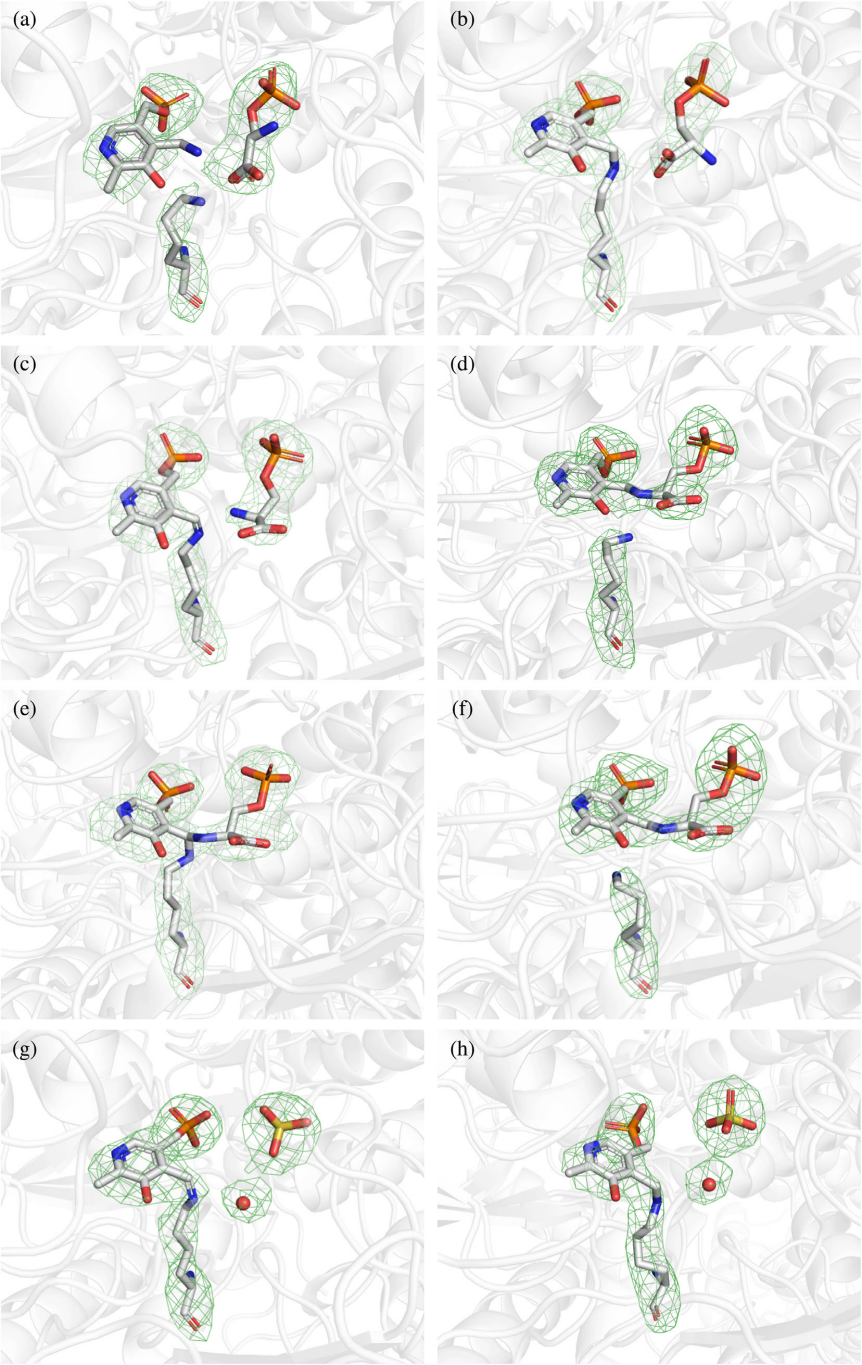
Different binding modes of ligands and cofactor observed in the eight active sites of *O*‐phosphoserine (OPS)‐phosphoserine aminotransferase structure. Polder omit maps are shown as green mesh contoured at 6 sigma, for the active site of each chain in the asymmetric unit. Chain A contains OPS, pyridoxamine phosphate, and free Lys200; chain B and C, contain OPS, and pyridoxal 5′‐phosphate (PLP) linked to Lys200 as internal aldimine; chains D and F, contain the external aldimine of OPS bound to the cofactor and free Lys200; in chain E, the gem‐diamine is clearly observed; chains G and H, contain PLP‐Lys200 as internal aldimine and the sulfate ion with a nearby water molecule.

After modeling the predominant reaction intermediate in the initial electron density, in some chains small blobs were left, suggesting the density might derive from the sum resulting from the co‐existence of different reaction intermediates. We used polder OMIT maps (Liebschner et al., 2017) to confirm the differences observed in the initial maps, and to improve the model building and refinement. The polder maps obtained for the final model are shown in Figure 6. Notably, in chains A, B, and C the OPS moiety occupies the substrate binding site with no covalent binding to the cofactor, which is in the PMP form in chain A and in the PLP form in chains B and C. Usually, substrate inhibition in transaminases involves substrate binding to the incorrect catalytic intermediate. The inhibition constant estimated for OPS and the high concentration of OPS used in soaking experiments (see Table 1) thus agree with the binding to the PMP‐PSAT structure. In chains D and F, the OPS‐PLP external aldimine is present, while in chain E the geminal diamine formed by Lys200, PLP, and OPS could be modeled.

This latter intermediate was also observed in the co‐crystallized OPS‐PSAT complex of *A. thaliana* (Sekula et al., 2018). In all these chains, the phosphate group of OPS replaces the sulfate anion modeled in the substrate‐free structure. In the other two chains (G and H) not enough density was present to model OPS. On the contrary, we could well fit the sulfate anion, which indeed occupies the phosphate binding site of the substrate, near the internal aldimine adduct in both chains.

**FIGURE 7:**
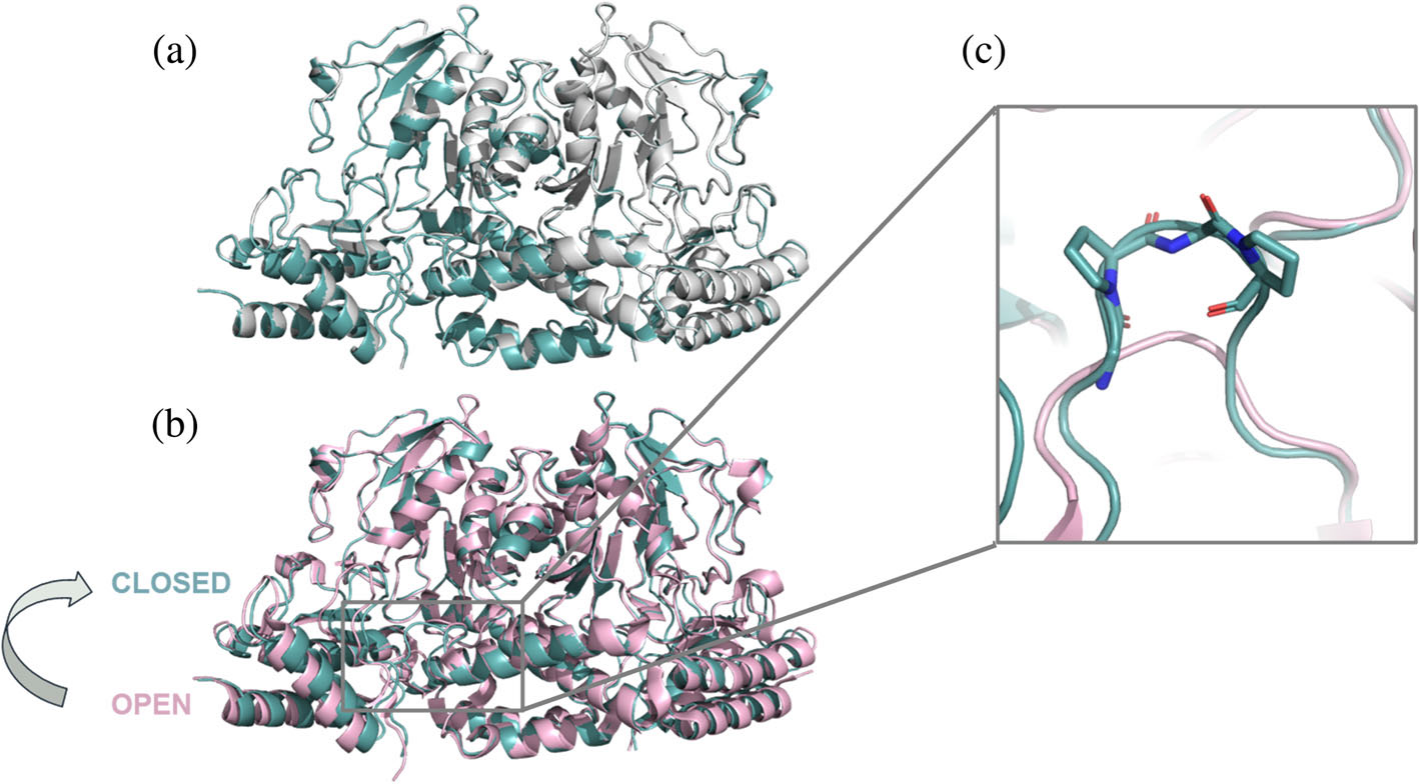
Comparison between the structures of human phosphoserine aminotransferase (PSAT). (a) Overlay between substrate‐free (cyan) and *O*‐phosphoserine‐bound (white) human PSAT solved in this work; (b) Overlay between substrate‐free human PSAT (cyan) and 3E77 (pink); (c) Active site close‐up of panel (b) showing the different N‐terminal loops, with the Pro‐Gly‐Pro motif highlighted in sticks mode.

Comparing the structures of the PSAT‐PLP/PMP dimer with that of PSAT bound to OPS, the main chains overlap quite well: no open–close movements were detected and both structures adopted a closed conformation (Figure 7a). While this might be expected for the OPS‐bound form, such an observation was surprising in the case of the substrate‐free form.

We performed a DALI (Holm & Rosenström, 2010) structural comparison against the PDB and the top 15 structures in the list are all PSAT (Table S6). Not surprisingly, the closest structure is that of the 3E77 human PSAT with an RMSD of 0.6 Å, indicating that the two models do not superimpose perfectly (Figure 7b). The present structure shows a closed conformation compared with 3E77, with the small domain moving of about 2 Å and with the 331–341 loop adopting a closed conformation, similar to the one observed in the OPS‐bound structure of *A. thaliana* (Sekula et al., 2018). The larger difference occurs at the level of the N‐terminal coil, which in 3E77 is much shorter due to the substitution of the first 16 amino acids with a sequence derived from cloning (Figures 7c and S8). The present structure demonstrates that also in the human enzyme, the N‐terminal coil, containing the highly conserved XGPY motif, forms a type IV turn that narrows the entrance to the active site and likely affects the activity of the protein, as shown for the *E. histolytica* enzyme (Singh et al., 2019).

### 2.5 Active site

The active sites are located at the monomer–monomer interface with the two cofactors placed about 30 Å apart, making several interactions with the backbone and side chains of the surrounding amino acids from both chains (Figure 5). The cofactor, either in the PLP‐Lys200 or in the PMP form, strongly interacts by pi stacking with the indole ring of Trp107 at distances ranging from 3.76 to 4.07 Å for the PLP‐Lys, and from 4.02 to 4.10 Å for the PMP moiety (Figure 5). The O and N atoms of the pyridine ring make hydrogen bonds with the side chains of Thr156 and Asp176, respectively. The phosphate group of the cofactor is kept in place by hydrogen bonds with the side chains of Gln199, Asn241*, and Thr242* and the main chains of Gly79, Cys80, and Thr242* (* refers to residues of the nearby subunit). The active site, in agreement with previous findings on PSATs from different organisms, is positively charged to better accommodate the negatively charged substrates. The surface electrostatic potential (Figure S9) is similar to that of other PSAT structures (Coulibaly et al., 2012), but with respect to that of the previously deposited human enzyme (3E77), the positively charged area is smaller, likely due to the partial closure of the active site associated to the presence of a full‐length N‐terminal sequence.

In the active sites of the substrate‐free form, close to each PLP/PMP moiety, a sulfate ion is clearly visible in the electron density forming salt bridges with the side chains of His44* and Arg45*, and with His335 (Figure 3e). Arg336, which is reported to be part of the phosphate binding site (Battula et al., 2013), is not always close enough to form strong hydrogen bonds. A sulfate ion was also found in the corresponding site in the structure of PSAT from *M. tuberculosis* (Coulibaly et al., 2012). The presence of sulfate is probably due to its high concentration in the crystallization medium; nevertheless, it mimics the binding of the phosphate moiety of the natural substrate.

**FIGURE 8:**
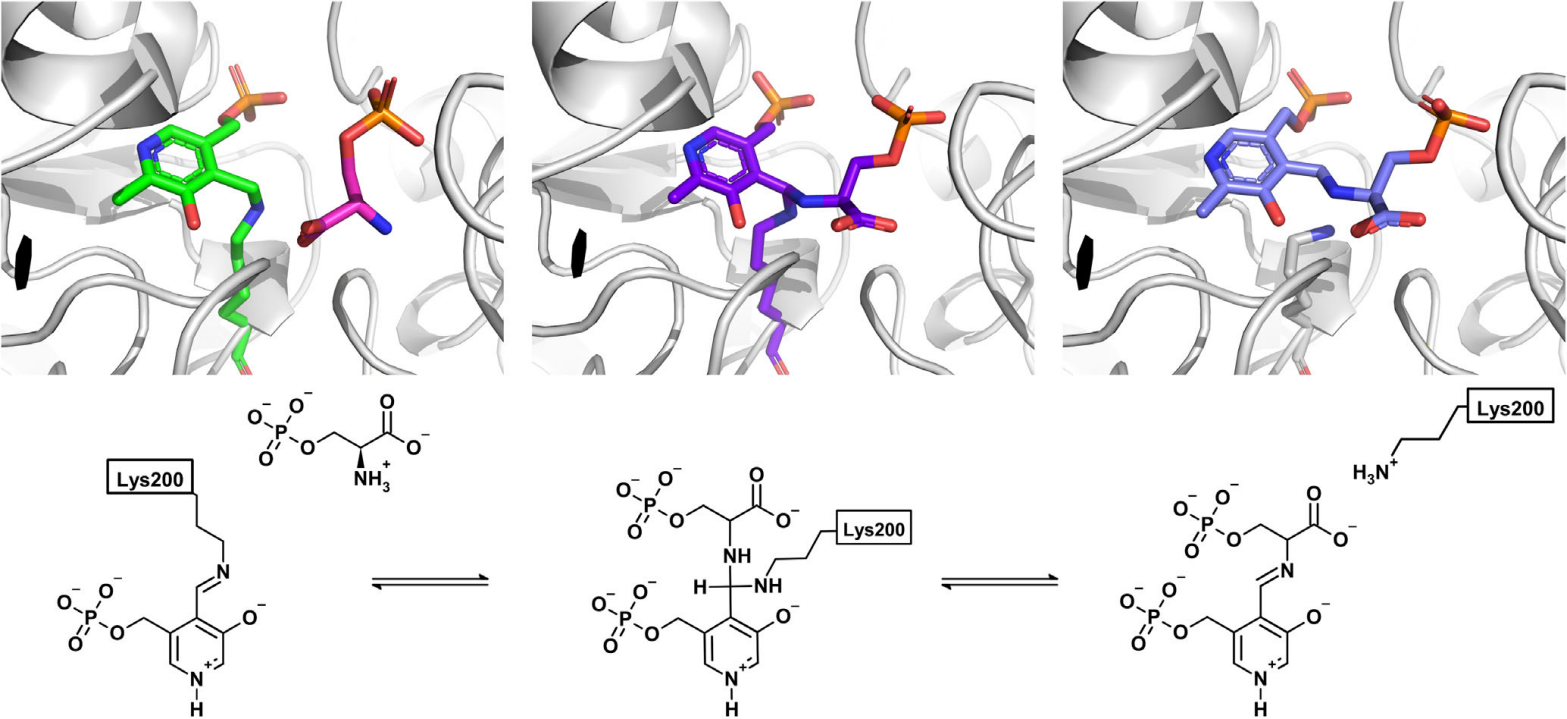
Snapshots of PSAT active site during the reaction with *O*‐phosphoserine (OPS). (a) Internal aldimine and unreacted OPS; (b) Geminal diamine; (c) External aldimine.

In the OPS‐soaked structure, in all the sites where OPS is found, the sulfate atom is replaced by the phosphate group of OPS that forms salt‐bridges with His44*, Arg45*, His335, and with Arg336 (Figure 3). Notably, in chains B and G, Arg336, which is located at the active site entrance, has a less clear electron density map, probably due to the acquisition of multiple orientations in the crystal. Another anchoring point for the substrate is given by Arg342, which makes salt bridges to the OPS carboxylate. Also, in the gem‐diamine case, the carboxylic group of OPS faces towards NH1 and NH2 of the guanidinium group of Arg342, making ionic bonds, while the binding with OPS to the internal aldimine involves only one oxygen, as the other one is facing towards the PLP moiety (Figure 3f and Table S7). The OPS carboxylate is also forming a hydrogen bond with the indolic nitrogen of Trp107. The cofactors, either in the PLP‐Lys or in the PMP forms, make similar interactions as described for the substrate‐free structure. In chain E, both PLP and OPS interact with the same residues as described above, with the additional formation of the geminal diamine between Lys200, PLP, and OPS. The same happens in chains D and F, where the OPS‐PLP external aldimine is present (Figures 4, 6, and 8c). Comparing all active sites of the four dimers among the two structures, where different cofactor and ligand binding poses were detected, we noticed little movements of the side and main chains (Table S7).

## 3 DISCUSSION

This study is the first detailed structural and functional characterization of human PSAT. While the overall picture agrees with published results on PSAT from different organisms, some specific features of the human ortholog emerged.

Human PSAT, consistently with the enzyme from other species, follows a ping–pong mechanism, with substrate inhibition, also observed for the bovine enzyme (Basurko et al., 1999). However, the inhibition constants are higher than the *in vivo* concentrations for the various substrates, thus a physiological and/or regulatory function of this inhibition is unlikely. The *K*_m_ values calculated in this work agree with those measured for the enzymes from bovine liver and *Entamoeba* (Ali & Nozaki, 2006; Basurko et al., 1999; Table S8), while the parameters estimated for PSAT from *A. thaliana* and sheep differ from those of the human ortholog. The *k*_cat_/*K*_m_ for PHP was among the highest ever reported for a transaminase; for comparison, the aspartate aminotransferases from chicken (Azzariti et al., 1998) and from water buffalo (Shahid Nadeem et al., 2011) showed *k*_cat_/*K*_m_ values in the order of 5 × 10^5^ M^−1^ s^−1^ towards the substrate α‐KG. The human tyrosine aminotransferase showed *k*_cat_/*K*_m_ values of 4.3 × 10^5^ and 4.6 × 10^4^ M^−1^ s^−1^ towards the substrates α‐KG and hydroxyphenyl pyruvate, respectively (Sivaraman & Kirsch, 2006). The *K*_m,3‐PHP_ and *K*_m,OPS_ are significantly higher than the in vivo substrate concentration (Table 1), suggesting that PSAT works under *k*_cat_/*K*_m_ regime in the cell and is thus a sensor for the levels of 3‐PHP, regulating its activity based on the availability of the glycolysis‐derived substrate. OPS formation by PSAT is 300‐fold more efficient than the reverse reaction and, considering a *K*_eq_ of about 10, the equilibrium of the whole pathway is shifted towards product formation, counteracting the unfavorable equilibrium of the first reaction catalyzed by PHGDH in the PP (Murtas et al., 2020; Murtas et al., 2021). Notably, human PSAT activity is strongly stimulated by increasing the ionic strength, that is, by 200 mM KCl.

PSAT is catalytically active also in the crystal form. Indeed, after quick soaking of the crystals in 50 mM OPS, different reaction intermediates, including the catalytic *gem*‐diamine and external aldimine, could be identified. The carboxylate group of the substrate makes a salt bridge with Arg342, whereas the phosphate group is anchored by ionic interactions to His44*, Arg45*, His335, and Arg336 which constitute the phosphate binding site (Figure 3 and Table S7). These interactions are retained in the *gem*‐diamine intermediate and in the external aldimine. In chains B and G, Arg366 has a poor electron density, suggesting a higher flexibility of this residue, which is located at the active site entrance. Interestingly, in the 3E77 structure, Arg366 is not visible in the electron density and the side chain has not been modeled. In our substrate‐free structure, where a sulfate ion occupies the phosphate binding site of the substrate, the electron density for Arg336 varies between the different chains, sometimes clearly interacting with the ion. In general, we suggest that this residue is flexible and might have an important role in locking the substrate during the reaction. PLP is close to Trp107, which likely plays the same stabilizing role on the cofactor orientation and position observed for the orthologous protein from *E. histolytica* and *Trichomonas vaginalis* (Mishra et al., 2012; Singh et al., 2016). The structure solved in this work contains the same N‐terminal loop that covers the active site in most PSAT structures. This loop plays a pivotal role in the activity of PSAT and was proposed to influence the stability of the dimer (Singh et al., 2019). Interestingly, this loop contains a Pro residue (Pro14 in the human sequence) in *cis* conformation, which favors the folding of the N‐terminus into a hairpin conformation that inserts in the active site groove. This Pro belongs to a well‐conserved motif, the XGPY sequence (Singh et al., 2019), which was absent in the 3E77 structure (Figure S8).

The substrate‐free structure well superimposes with the structure of OPS‐bound PSAT, with an RMSD of 0.143 Å, and both show a closed conformation. On the contrary, the superposition of our structures with the deposited structure of human PSAT reveals main differences in the small domain that, together with the N‐terminal loop, contributes to form a close pocket. In the case of substrate‐free PSAT, a sulfate ion is visible in the active site and its position is superimposable with that of the phosphate moiety of OPS. We thus conclude that sulfate and phosphate itself can bring about most of the conformational changes needed for the open‐to‐closed transition. We believe that the structures presented here are good models of the human PSAT in a conformation that is proficient for substrate binding, a useful tool to clarify the structure–function relationships of this enzyme under physiological as well as pathological conditions related to genetic mutations.

## 4 MATERIALS AND METHODS

### 4.1 Expression and purification of human PSAT

The gene coding for human PSAT‐β (*psat*) was cloned in the CpoI site of a modified pET28b plasmid in frame with a sequence encoding a hexahistidine tag (Donini et al., 2009). The expression was carried out at 20°C in *E. coli* BL21(DE3) tuner cells (Novagen®, Merck, Darmstadt, Germany) by addition of 0.2 mM isopropyl‐β‐D‐1‐thiogalactopyranoside. The protein in a buffer containing 25 mM Tris pH 8.0, 300 mM NaCl, 0.2% (vol/vol) Tween‐20, 50 μM PLP, 1 mM TCEP, 1 mg/mL lysozyme, and protease inhibitors was purified by IMAC (Talon superflow–Cytiva™) using an FPLC system (Akta Prime ‐ GE™). The protein was dialyzed against 25 mM Tris pH 8, 300 mM NaCl, 1 mM TCEP and 4 μM PLP, concentrated, flash frozen in liquid nitrogen, and stored at −80°C. The final yield of purification was 70 mg per liter of culture broth. The protein for crystal preparation was purified using the same protocol reported above using a different buffer system (30 mM HEPES, 100 mM KCl, 4 μM PLP, and 1 mM DTT pH 7.5) and then dialyzed against 50 mM sodium phosphate, 300 mM NaCl and 1 mM TCEP pH 8.0. The same preparation was used for both spectroscopic characterization and crystallization experiments. Prior to crystallization, PSAT was further purified on a Superdex 75 16/60 column (Cytiva) equilibrated with 20 mM HEPES pH 7.5, 300 mM NaCl, 1 mM TCEP. The final protein sample was concentrated to 8–10 mg/mL and immediately used for the crystallization trials. Pure PLP‐forms and PMP‐forms of PSAT were prepared by adding either 0.11 mM PHP or 20 mM L‐Glu to the protein solution in 25 mM Tris, 300 mM NaCl pH 7.4. The reaction was followed spectrophotometrically. Upon reaction completion, PSAT was immediately subjected to extensive ultrafiltration against 20 mM potassium phosphate pH 7 to remove traces of substrates and products.

### 4.2 Absorption spectroscopy

Absorption spectra were collected using a Cary4000 spectrophotometer (Agilent Technologies®). Protein concentration was calculated using an extinction coefficient at 280 nm of 35,870 M^−1^ cm^−1^ (calculated by the ProtParam server). Absorption spectra of PSAT at different pH values were collected at room temperature in the multicomponent buffer P (50 mM MES, 50 mM HEPES, 50 mM bicine, and 100 mM NaCl). Different pH values were obtained by mixing buffer P at acidic pH with different volume ratios of the same buffer titrated to pH 9.5 with NaOH. Each spectrum was corrected for the baseline contribution. The absorbance values at 408 nm and 344 nm were plotted as a function of pH and data were fitted to Equation (1) to calculate the p*K*_a_ of the cofactor aldimine (Montioli et al., 2013):

(1) $$\mathrm{Abs}=y_{0}+\frac{\mathrm{Abs}}{1+10^{pK_{a}-\mathrm{pH}}}$$

where Abs is the absorbance at either 344 nm or at 408 nm and *y*_0_ is an offset.

### 4.3 Fluorescence spectroscopy

Fluorescence spectra of PSAT were collected in buffer P at 20°C using a Fluoromax spectrofluorometer (HORIBA‐Jobin Yvon®). Slits were set for optimal signal‐to‐noise ratio. Emission spectra of the cofactor were collected upon excitation at 330 nm and 344 nm at pH 5.8 and pH 9.5, respectively (as determined by fluorescence excitation spectra; Figure S2). Emission spectra of tryptophan were collected upon excitation at 298 nm at both pH values. The effects of potassium chloride and potassium phosphate on the emission of tryptophan residues were evaluated in a solution containing 4 μM PSAT in 50 mM HEPES pH 7 in the absence and presence of either 0.2 M potassium chloride or 0.2 M potassium phosphate.

### 4.4 Circular dichroism and thermal stability

The far‐UV and near‐UV circular dichroism spectra of PSAT were collected using a Jasco® 1500 spectropolarimeter equipped with a Peltier system for the temperature control. The far‐UV spectra were collected on 5 μM PSAT solutions in 20 mM potassium phosphate pH 7, 20°C in a 0.1 cm optical pathlength cuvette. Each spectrum is the average of three acquisitions and was corrected for the buffer contribution. The melting curves (at 222 nm) were collected from 25°C to 80°C under the same conditions, with a data pitch of 1°C, a ramp rate of 5°C/min, a bandwidth of 1 nm and a digital integration time of 1 s. Data were fitted to (Equation 2; Huynh & Partch, 2015):

(2) $$\theta =\theta_{0}+A\;\left[\frac{f}{1+e^{\frac{T-T_{m1}}{k_{1}}}}+\frac{1-f}{1+e^{\frac{T-T_{m2}}{k_{2}}}}\;\right]$$

where *θ* is the ellipticity at 222 nm, *θ*_0_ is an offset, *A* is the amplitude, *f* is the fractional abundance of the first phase, *T* is the temperature in °C, *T*_*m*1_ and *T*_*m*2_ are the melting temperatures of the two phases and *k*_1_ and *k*_2_ are the slope of the two phases. The near‐UV circular dichroism spectrum of PLP‐PSAT was collected on 40 μM PSAT solutions in 20 mM potassium phosphate pH 7, 20°C in a 0.2 cm optical pathlength cuvette.

### 4.5 Preparation of 3‐PHP

3‐Phosphohydroxypyruvic acid was obtained from the dimethyl ketal (eNovation Chemicals®), as described by Ballou and Hesse (1956). The amount of 3‐PHP was estimated enzymatically using human PHGDH and the NADH extinction coefficient (6220 M^−1^ cm^−1^). The yield of the reaction was ~85%.

### 4.6 Activity assays

The activity assays for the determination of the kinetic parameters of human PSAT were performed in buffer A (50 mM HEPES pH 7, 100 mM KCl, 1 mM DTT, 0.170 mM PLP) using a Cary4000 spectrophotometer (Agilent technologies®). The assay mixture, without the enzyme, was incubated in the cuvette holder for 2 min at 37°C before reading the baseline absorbance at 340 nm for 1–2 min. The reactions were then started by adding PSAT, and the initial velocity was calculated using an extinction coefficient for NADH of 6220 M^−1^ cm^−1^, after subtracting the rate of the incubation phase. The forward reaction was followed by coupling the production of α‐KG with the reaction of glutamate dehydrogenase (GDH; type II from bovine liver–MERCK®; one unit per assay), in the presence of 81 nM PSAT, 0.1 mM NADH, 32 mM NH_4_Cl (Basurko et al., 1999), 3‐PHP ranging from 0.011 to 0.11 mM and L‐Glu ranging from 0.4 to 80 mM. The rate depended linearly on PSAT concentration in the 21–167 nM range (Figure S3), which corresponds approximately to an initial velocity between 0.3 and 3 μM/s. The reverse reaction was followed by coupling the production of 3‐PHP with 8 mU of human recombinant PHGDH in the presence of 337 nM PSAT, 0.1 mM NADH, α‐KG ranging from 0.1 to 10 mM, and L‐OPS ranging from 0.03 to 1 mM. The rate depended linearly on the transaminase concentration in the 167–340 nM range (Figure S3), which corresponds approximately to an initial velocity between 1 and 3 μM/s. Recombinant human PHGDH was produced as reported in Murtas et al. (2021).

The dependence of the initial rate on the concentration of both substrates was fitted to Equation (3) that account for a ping–pong mechanism with substrate inhibition (Cook, 2007):

(3) $$v_{0}=\frac{V_{\max}\left[A\right]\left[B\right]}{K_{m\;\left(B\right)}\left[A\right]\left(1+\frac{\left[A\right]}{K_{i\left(A\right)}}\right)+K_{m\;\left(A\right)}\left[B\right]\left(1+\frac{\left[B\right]}{K_{i\left(B\right)}}\right)+\left[B\right]\left[A\right]}$$

where *v*_0_ is the initial velocity; *V*_max_ is the rate at substrate saturation; [*A*] is the concentration of either L‐Glu or OPS for the forward and reverse reaction, respectively; [*B*] is the concentration of either 3‐PHP or a‐KG for the forward and reverse reaction, respectively; *K*_*m*(*A*)_ and *K*_*i*(*A*)_ are the Michaelis–Menten constant and the inhibition constant for substrate A, respectively; *K*_*M*(*B*)_ and *K*_*i*(*B*)_ are the Michaelis–Menten constant and the inhibition constant for substrate B, respectively.

Kinetic parameters were also determined by fitting individual kinetic traces of substrate consumption to an integrated Michaelis–Menten equation (Equation 4; Goličnik, 2013; Schnell & Mendoza, 1997):

(4) $$\left[S\right]\left(t\right)=K_{M}W\left(\frac{S_{0}}{K_{M}}\;e^{\left(S_{0}-V_{\max}t/K_{M}\right)}\right)$$

where *K*_M_ is the Michaelis–Menten constant, *W* is the omega function, *S*_0_ is the initial concentration of substrate, *V*_max_ is the maximal activity and *t* is the time in minutes.

Haldane equation for a ping–pong mechanism (Equation 5) was used to determine the *K*_eq_ using kinetic parameters (Cleland, 1982; Noor et al., 2013):

(5) $$K_{\mathrm{eq}}=\frac{K_{M\left(a-\mathrm{KG}\right)}\;K_{M\left(L-\mathrm{OPS}\right)}}{K_{M\;\left(3-\mathrm{PHP}\right)}\;K_{M\;\left(L-\mathrm{Glu}\right)}}\;{\left(\frac{k_{\mathrm{cat}\;\left(\text{for}\right)}}{k_{\mathrm{cat}\;\left(\mathrm{rev}\right)}}\right)}^{2}$$

where *K*_eq_ is the equilibrium constant, *k*_cat(for)_ is the maximal rate for the forward reaction, *k*_cat(rev)_ is the maximal rate for the reverse reaction and *K*_M(α‐KG)_, *K*_M(3‐PHP)_, *K*_M(L‐Glu)_, and *K*_M(L‐OPS)_ are the Michaelis–Menten constants for α‐KG, 3‐PHP, L‐Glu, and L‐OPS.

The determination of the initial velocity of PSAT at different pH values was performed in buffer P.

The activity of PSAT with CSA was measured in buffer A with the addition of 0.1 mM NADH, 0.11 mM 3‐PHP, 4–8 units of lactate dehydrogenase from bovine heart (MERCK®), CSA ranging from 10 to 265 mM and 330 nM PSAT (Furumo & Kirsch, 1995).

The IC_50_ for phosphate was estimated by measuring the activity of PSAT (reverse reaction to avoid any artifacts due to the presence of NH_4_Cl in the assay mixture for the forward assay) at different concentrations of potassium phosphate, ranging from 0.01 to 290 mM. Initial velocity at different concentrations of potassium phosphate was measured in the presence of substrates concentrations equal to the *K*_m_ for both OPS and α‐KG (i.e., 0.03 mM and 0.5 mm, respectively). Data were fitted to Equation (6).

(6) $$\frac{v_{i}}{v_{0}}=\frac{{\mathrm{IC}}_{50}\;}{\left[{\mathrm{KP}}_{i}\right]+{\mathrm{IC}}_{50}}$$

where, *v*_0_ is the initial velocity in the absence of phosphate and *v*_i_ is the initial velocity at a given phosphate concentration.

### 4.7 Estimation of the equilibrium constants

The equilibrium constant for the conversion of L‐Glu/3PHP to α‐KG and L‐OPS was estimated in buffer A at 37°C using a protocol adapted from Merrill et al. (1981) Briefly, 2 mM OPS and 2 mM α‐KG were incubated for 3 min at 37°C and the reaction was started by adding 3 μM PSAT. The mixture was sampled over time and the reaction was stopped with 1% trichloroacetic acid. The concentrations of α‐KG and 3‐PHP at each time point were estimated enzymatically after neutralization with potassium hydroxide. Quantification reactions were performed in buffer A with the additional presence of either 0.22 mM NADH, 32 mM NH_4_Cl, and 0.25 U of GDH for the determination of α‐KG or 0.22 mM NADH and 1 mU of PHGDH for the determination of 3‐PHP. The reaction was allowed to proceed until completion (about 45 min) and the concentrations of α‐KG and 3‐PHP were calculated from the ΔOD_340 nm_ of NADH (using *ε*_340_ = 6220 M^−1^ cm^−1^).

### 4.8 Crystallization, data collection, data reduction, structure determination, refinement, and final model analysis

Needle‐like crystals were initially obtained under multiple conditions but diffracted poorly (8–11 Å). After optimization, slightly thicker and bigger crystals were obtained that were unreproducible, very brittle, and sensitive to cryoprotectants. These were grown in 7 days from hanging drops, mixing 1 μL of PSAT with 1 μL of 0.2 M Na_2_SO_4_, 0.1 M Bis Tris Propane, pH 6.5, or 7.5, 20% PEG 3350. Crystals were dipped in 30% MPD cryoprotectant for a few seconds before freezing. The OPS complex structure was obtained by soaking crystals for a few seconds in 50 mM OPS in the cryoprotectant solution. Data were collected at 100 K at the XDR2 beamline of the Elettra synchrotron in Trieste (Lausi et al., 2015) using a 0.9717 Å wavelength. The datasets were processed with AutoPROC (Vonrhein et al., 2011) and STARANISO 2.3.77 (Tickle et al., 2018). The structure was solved by molecular replacement with Phaser (McCoy et al., 2007) using as search model 3E77 (PDB ID). The initial model was refined through alternating cycles of manual model building in COOT (Emsley et al., 2010; Emsley & Cowtan, 2004) and automatic refinement using Phenix (version 1.18.2_3874). Data collection and refinement statistics are reported in Table S9.

### 4.9 Accession codes

Coordinates and structure factors were deposited in the PDB with accession numbers 8A5V (substrate‐free PSAT) and 8A5W (OPS‐bound PSAT).

## AUTHOR CONTRIBUTIONS

**Francesco Marchesani:** Formal analysis (equal); investigation (lead); visualization (equal); writing – original draft (equal); writing – review and editing (equal). **Erika Zangelmi:** Investigation (equal); writing – review and editing (equal). **Giulia Murtas:** Investigation (equal); writing – review and editing (equal). **Elisa Costanzi:** Formal analysis (equal); investigation (equal); visualization (equal); writing – original draft (equal); writing – review and editing (equal). **Raheem Ullah:** Formal analysis (equal); investigation (equal); writing – review and editing (equal). **Alessio Peracchi:** Conceptualization (equal); resources (equal); supervision (equal); writing – original draft (equal); writing – review and editing (equal). **Stefano Bruno:** Conceptualization (equal); resources (equal); writing – original draft (equal); writing – review and editing (equal). **Loredano Pollegioni:** Conceptualization (equal); funding acquisition (lead); resources (equal); supervision (equal); writing – review and editing (equal). **Andrea Mozzarelli:** Conceptualization (equal); resources (equal); supervision (equal); writing – original draft (equal); writing – review and editing (equal). **Paola Storici:** Conceptualization (equal); resources (equal); supervision (lead); visualization (equal); writing – original draft (lead); writing – review and editing (equal). **Barbara Campanini:** Conceptualization (equal); funding acquisition (lead); resources (equal); supervision (lead); writing – original draft (lead); writing – review and editing (equal).

## Supporting information


**Data S1:** Supporting information.

## Data Availability

The data that support the findings of this study are available from the corresponding author upon reasonable request.
