# Supplementary material for: L‐serine biosynthesis in the human central nervous system: Structure and function of phosphoserine aminotransferase
Source: Protein Sci. 2023 Apr 1;32(4):e4609. doi: 10.1002/pro.4609 (PMC10031235; doi:10.1002/pro.4609)
Supplement: Supplementary file 1 — Data S1: Supporting information. [file PRO-32-e4609-s001.docx]

**L-SERINE BIOSYNTHESIS IN THE HUMAN CENTRAL NERVOUS SYSTEM: STRUCTURE AND FUNCTION OF PHOSPHOSERINE AMINOTRANSFERASE**

Francesco Marchesani^1^, Erika Zangelmi^2^, Giulia Murtas^3^, Elisa Costanzi^4^, Raheem Ullah^4,5^, Alessio Peracchi^2^, Stefano Bruno^1^, Loredano Pollegioni^3^, Andrea Mozzarelli^6^, Paola Storici^4^, Barbara Campanini^1^

^1^ Department of Food and Drug, University of Parma, Parma, Italy

^2^ Department of Chemistry, Life Sciences and Environmental Sustainability, University of Parma, Parma, Italy

^3^ Department of Biotechnology and Life Sciences, University of Insubria, Varese, Italy

^4^ Protein Facility, Elettra Sincrotrone Trieste S.C.p.A., Trieste, Italy

^5^ Present address: Structural Biology Lab, NIBGE, Faisalabad, Pakistan

^6^ Institute of Biophysics, CNR, Pisa, Italy

Correspondence to:

Barbara Campanini, Department of Food and Drug, University of Parma, Parma, Italy. Email: [barbara.campanini@unipr.it](mailto:barbara.campanini@unipr.it)

Paola Storici, Protein Facility, Elettra Sincrotrone Trieste S.C.p.A., Trieste, Italy. Email: [paola.storici@elettra.eu](mailto:paola.storici@elettra.eu)

Running title: Biochemical characterization of human PSAT

**SUPPLEMENTARY MATERIALS**

1. **Substrate binding, thermal stability and spectroscopic features of human PSAT**


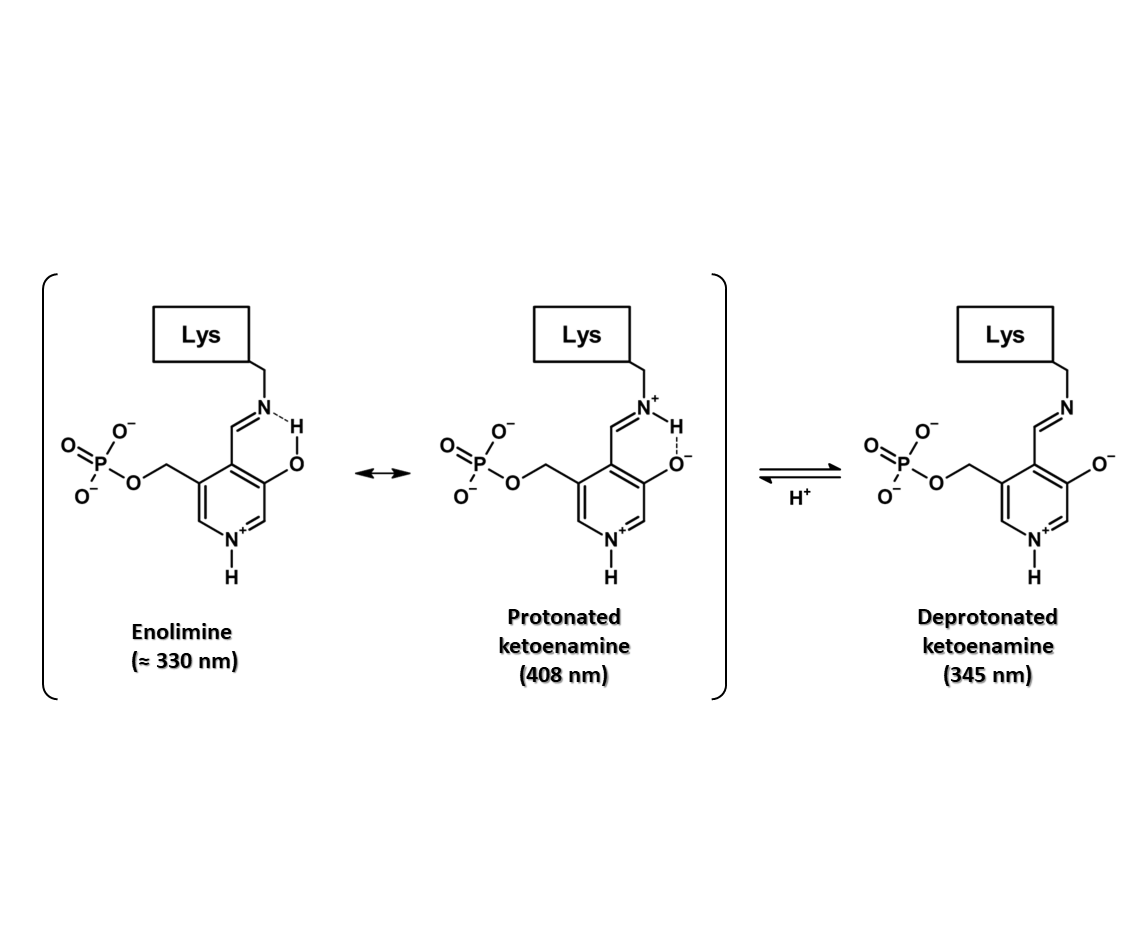


**Scheme S1**: Tautomeric equilibrium between the enolimine and the protonated ketoenamine forms of PLP followed by the deprotonation of ketoenamine tautomer. The wavelength of maximum absorption is indicated in brackets.

**
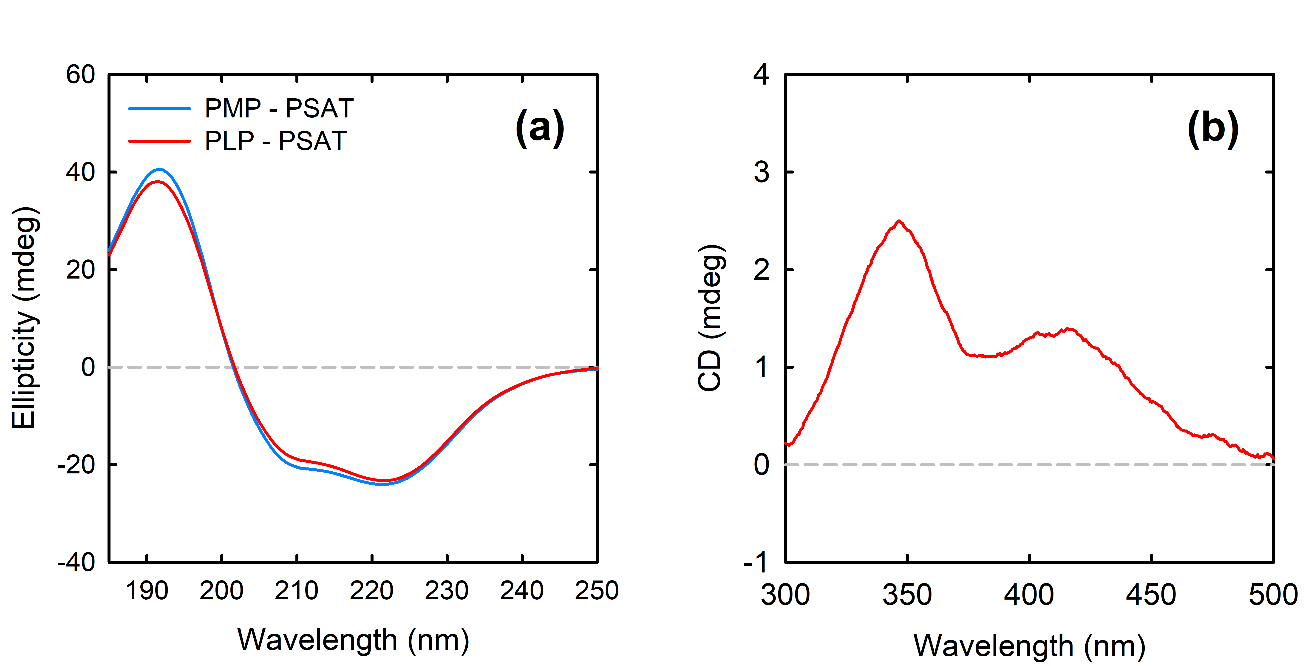
**

**Figure S1**: Circular dichroism spectra of PSAT; (a) Far UV CD spectra of 5 µM PMP-PSAT (blue line) or PLP-PSAT (red line); (b) Near UV spectra of 40 µM PLP-PSAT.

**
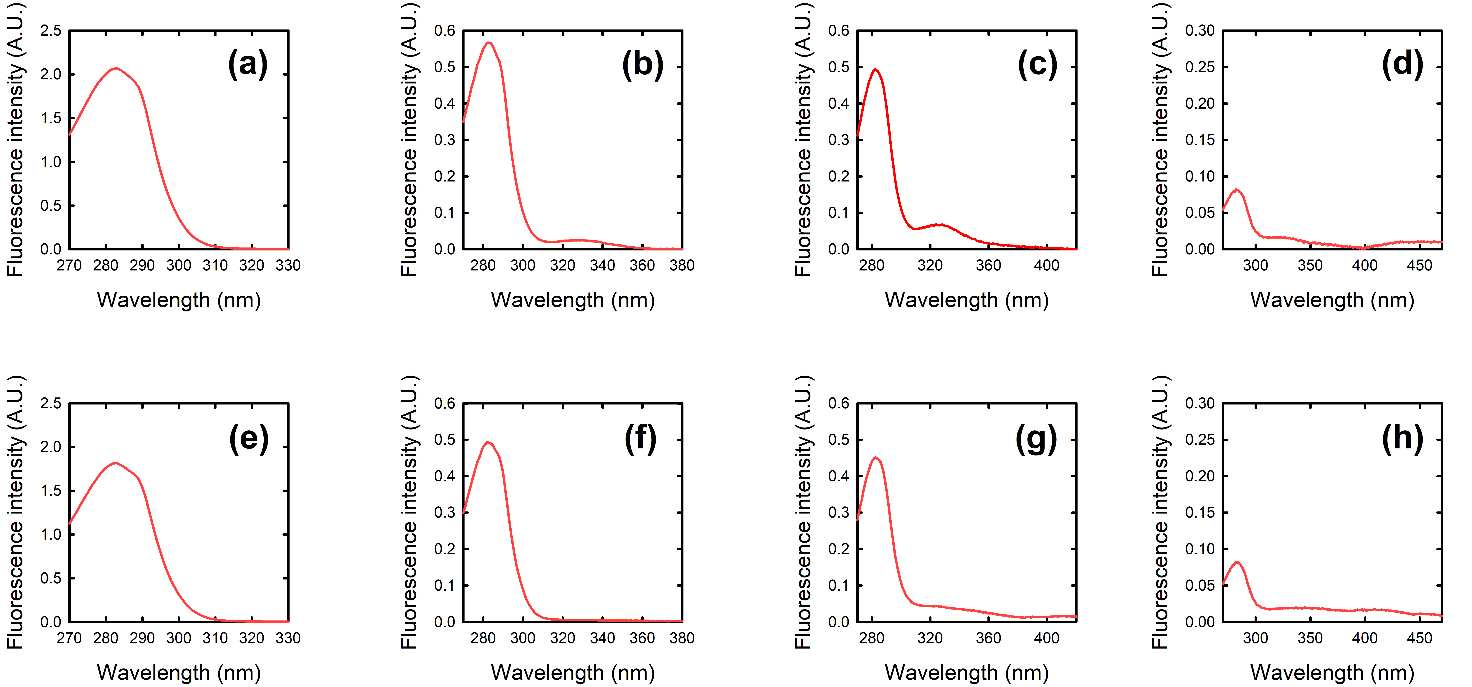
**

**Figure S2:** Excitation spectra of 5 µM PSAT at pH 5.8 ((a)-(d)) and pH 9.5 ((e)-(h)). (a)(e) Emission at 350 nm (slits 3 nm). (b) (f) Emission at 400 nm (slits 3 nm). (c) (g) Emission at 450 nm (slits 5 nm). (d) (h) Emission at 500 nm (slits 5 nm).

1. **Human PSAT efficiently catalyzes the transamination of 3-PHP using L-Glu as amino donor**


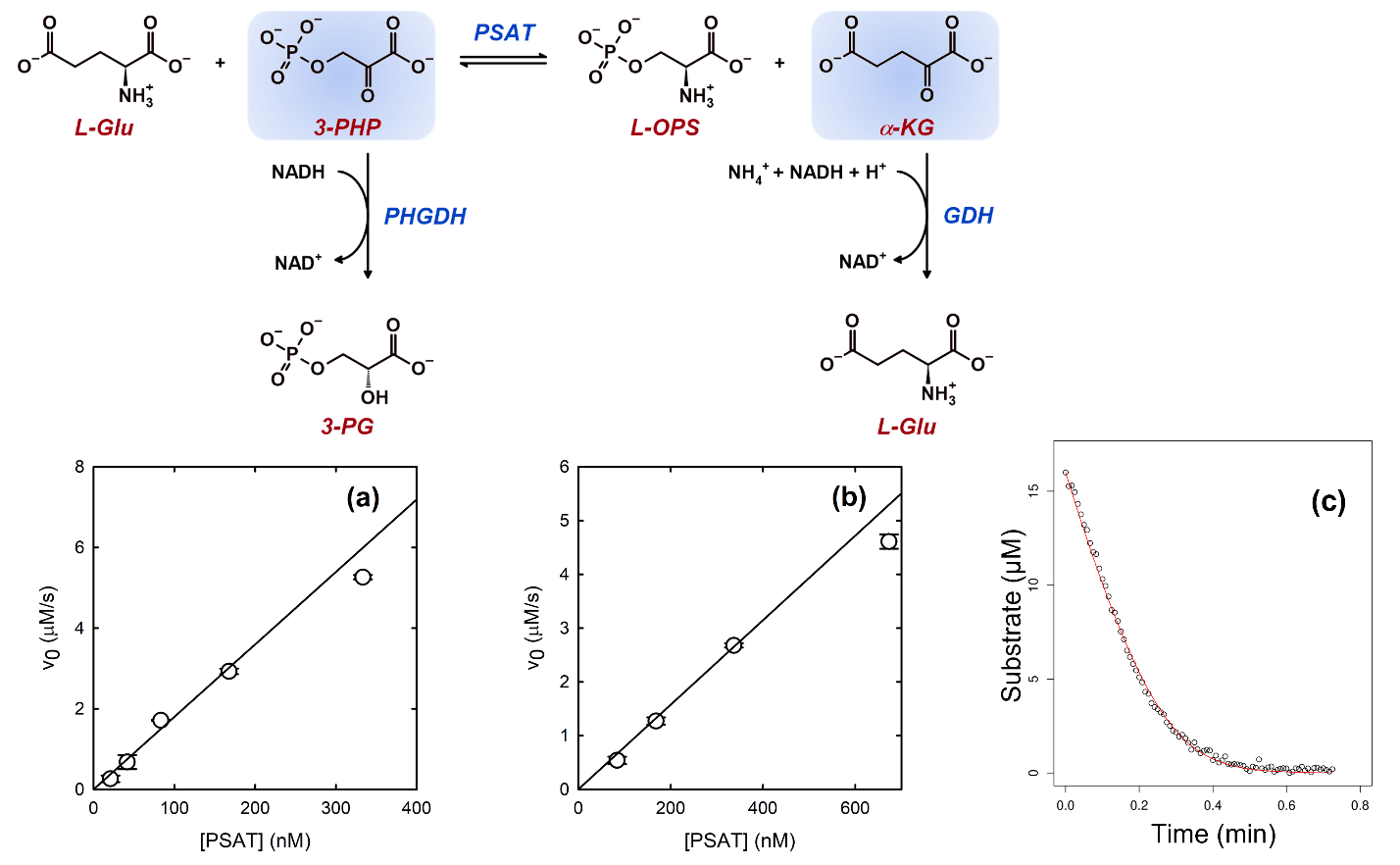


**Figure S3:** Activity assays for the determination of the catalytic parameters of PSAT. **Upper panel:** Scheme of the coupled assays for the detection of the forward and reverse reactions of PSAT. (a) Dependence of the initial velocity of the forward reaction catalyzed by PSAT on enzyme concentration in the presence of 20 mM L-Glu, 0.22 mM 3-PHP and 550 mU of GDH. (b) Dependence of the initial velocity of the reverse reaction catalyzed by PSAT on enzyme concentration in the presence of 0.7 mM L-OPS, 5 mM α-KG, and 8 mU PHGDH. (c) Kinetic trace of the forward reaction catalyzed by 81 nM PSAT in the presence of 20 mM L-Glu, 0.22 mM 3-PHP, and 1 U GDH. The line through the data points represents the fitting to the integrated Michaelis-Menten equation (eq.4) with K_m,3-PHP_ =6.9 ± 0.5 μM and k_cat_ =19 ± 0.7 s^-1^.

**Table S1:** Concentrations of α-KG and 3-PHP at different time points after triggering the reaction by adding PSAT at 37 °C to a solution containing 2 mM OPS and 2 mM α-KG.

| **Time (min)** | **[α-KG] (mM)** | **[3-PHP] (mM)** | **[α-KG] / [3-PHP]** |
| --- | --- | --- | --- |
| 10 | 1.96 ± 0.19 | 0.09 ± 0.02 | 22.5 ± 3.1 |
| 20 | 1.91 ± 0.02 | 0.13 ± 0.01 | 15.1 ± 1.3 |
| 30 | 1.93 ± 0.18 | 0.16 ± 0.01 | 12.1 ± 1.6 |
| 45 | 1.87 ± 0.05 | 0.16 ± 0.01 | 11.5 ± 1.1 |

**
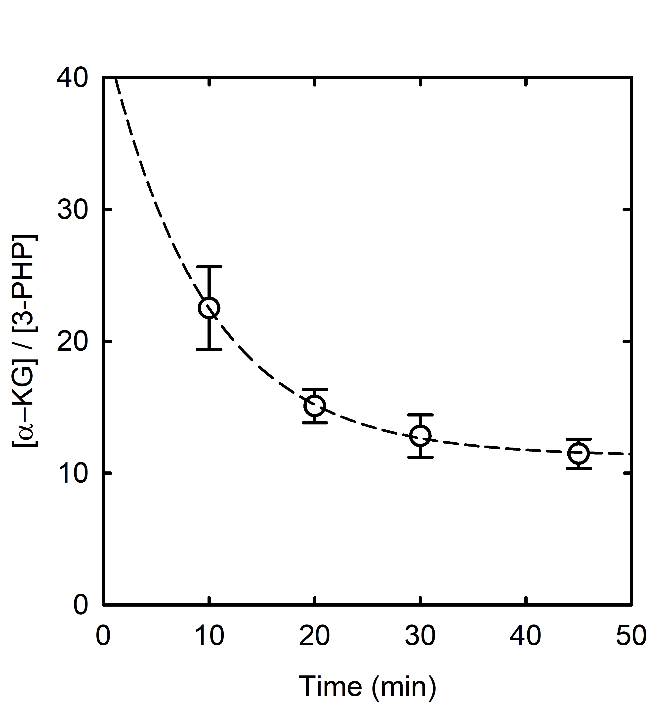
**

**Figure S4:** Determination of the equilibrium constant of the reaction catalyzed by PSAT. The ratio of α-KG and 3-PHP (see Table S1) is plotted as a function of time. The dashed line is the fitting of the data points with an exponential decay plus an offset. The y-value at infinite times obtained from the fitting, i.e. 11.3 ± 0.3, represents the K_eq_.

1. **Alternative substrates and activity modulators**


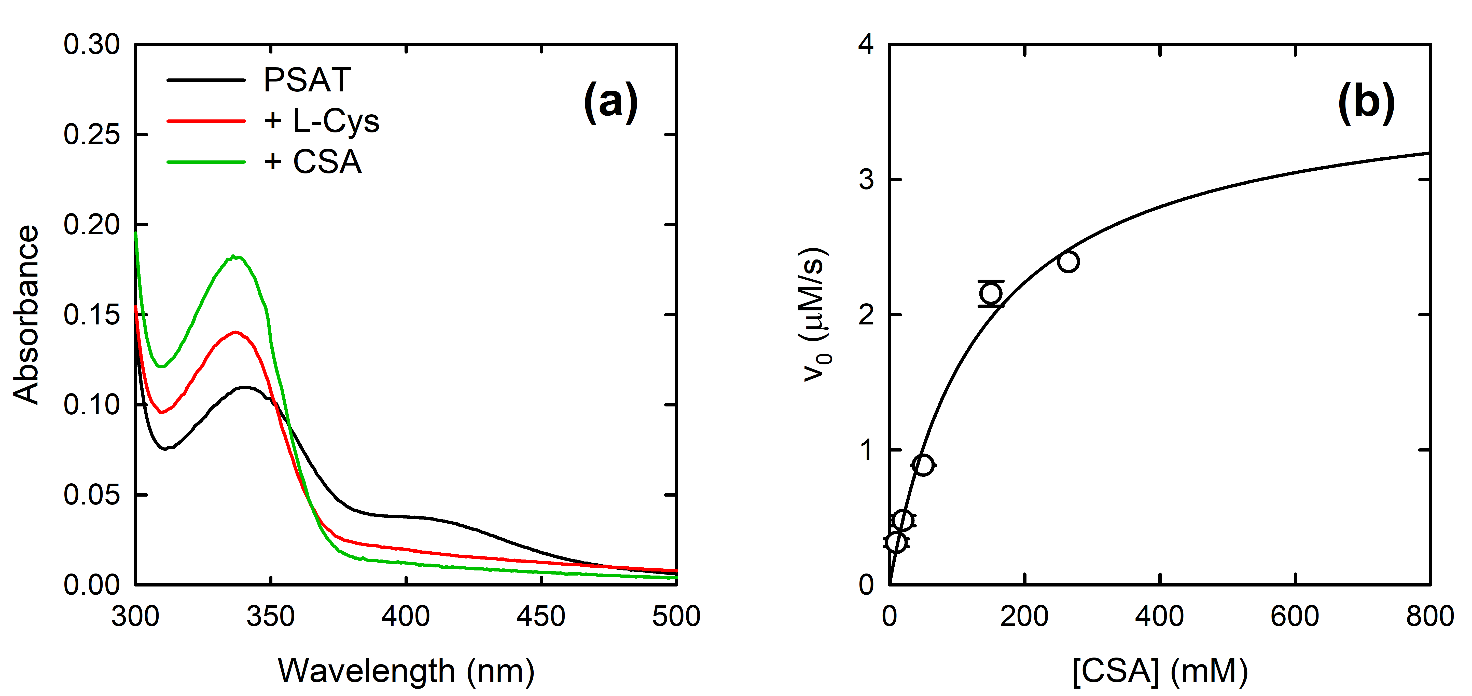


**Figure S5:** Characterization of the reaction of L-cysteine and L-cysteine sulfinate with PSAT. (a) Absorption spectra of 30 µM PSAT in 20 mM potassium phosphate pH 7.4 in the absence (black line) and presence of either 100 mM L-cysteine (red line) or 100 mM L-cysteine sulfinate (green line). (b) Dependence of reaction rate on CSA concentration in the presence of 0.11 mM 3-PHP, 8 U of lactate dehydrogenase. The line is the fitting of the data points to the Michaelis-Menten equation. The fitting gave large errors in the estimate of catalytic parameters. Hence, k_cat_/K_m_ was calculated by the slope of the initial part of the hyperbola as 58.1 M^-1^s^-1^.

**3.1 Effect of nucleotides and salts**

We deemed it interesting to investigate the effect of selected nucleotides on the activity of PSAT (**Figure S6a**) because the phosphorylated pathway uses a glycolytic intermediate as precursor for L-Ser and regulation by the energy status of the cell might be in play. Neither NAD^+^/NADH, nor ATP/AMP exerted any effect on the activity of PSAT, suggesting that a specific binding site for these molecules is not present on the protein and that binding to Cibacron blue-agarose reported for bovine PSAT^1,2^ is rather due to the presence of a large patch of positively charged residues at the active site entrance.

We further investigated the dependence of activity on the concentration of added salts initially on the forward reaction, but the presence of high NH_4_Cl concentrations required for the GDH reaction already saturates the effect (**Figure S6b**)**.** No effect of cations was apparent from these assays, since the activities measured in the presence of ammonium, sodium, potassium and magnesium chloride were all comparable. When the effect of salts at a 100 mM concentration was investigated on the reverse reaction a 4- to 5-fold increase in the reaction rate was observed (**Figure S6c**). We thus explored the effect of various halogen ions on the activity. NaCl, NaBr, NaI, and NaF increased the transamination rate, with NaF showing the less pronounced effect (**Figure S6d**). A specific binding site for halides was reported for *Bacillus alcalophilus* PSAT^3^ and an effect of halides was reported on the activity of the enzyme from *Trichomonas vaginalis*.^4^ However, in the case of human PSAT, this behavior likely mirrors a nonspecific effect of the ionic strength on the enzyme activity. Indeed, the chloride binding site is not conserved in PSAT from *Trichomonas vaginalis*.^4^


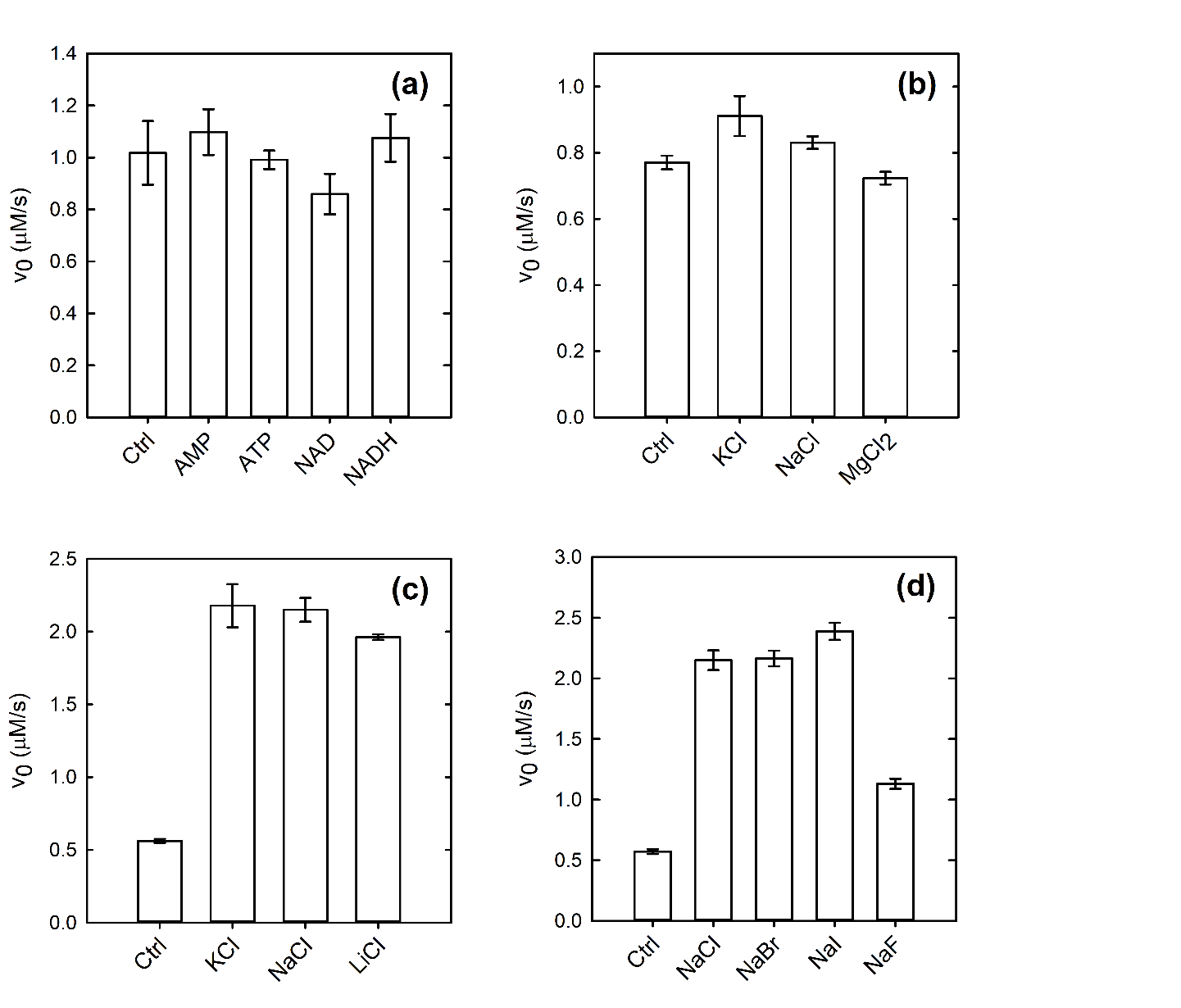


**Figure S6**: Effect of nucleotides and salts on PSAT activity. (a) Effect of nucleotides at 1 mM on the activity of 40 nM PSAT (forward reaction) in the presence of 20 mM L-Glu and 0.11 mM 3-PHP. (b) Activity of 40 nM PSAT (forward reaction) in the presence of 20 mM L-Glu, 0.11 mM 3-PHP and 100 mM of different salts. All the experiments were carried out in the presence of 32 mM NH_4_Cl, essential for the activity of the coupled enzyme GDH. (c) Activity of 336 nM PSAT (reverse reaction) in the presence of 1 mM L-OPS, 5 mM α-KG and 100 mM of different salts. (d) Effect of different sodium halides on the activity of 336 nM PSAT (reverse reaction) in the presence of 1 mM L-OPS and 5 mM α-KG.

1. **Overall structure of substrate-free and OPS-bound PSAT**


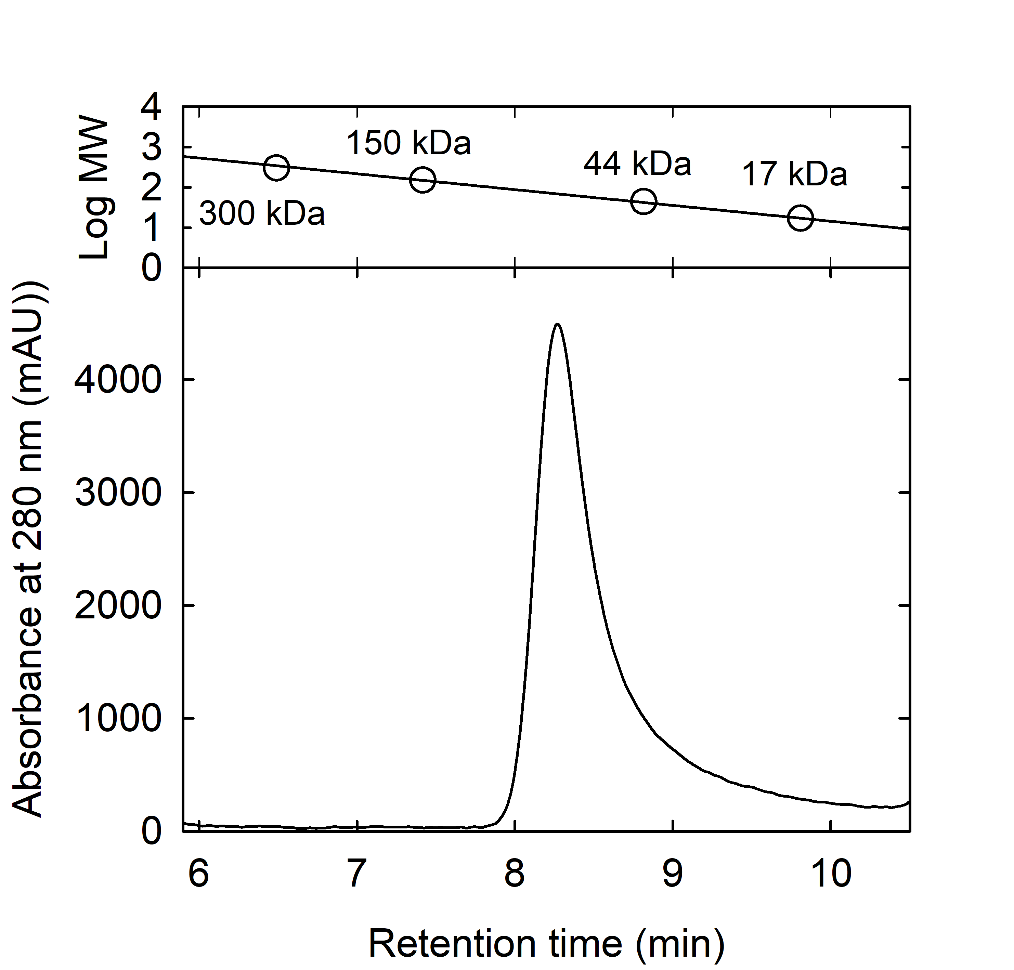


**Figure S7**: Chromatogram of 5 µM PSAT eluting from a BioSep-SEC-2000 column (300 mm, 1.50 mm, 5 μM Phenomenex) in isocratic elution mode with a mobile phase composed of 50 mM K_2_HPO_4_, 300 mM NaCl pH 7 at a flow rate of 1 mL/min. Inset: Calibration curve built with a Column Performance Check Standard, Aqueous SEC 1 (Phenomenex), including myoglobin (17 kDa), ovalbumin (44 kDa), IgG (150 kDa), and IgA (300 kDa). Based on the calibration curve, PSAT has an apparent MW of 69 kDa.

**Table S2.** RMSD between the 4 different PSAT dimers present in the asymmetric unit in the substrate-free structure.

| **Dimer** | **AB** | **CD** | **EF** | **GH** |
| --- | --- | --- | --- | --- |
| **AB** | 0.000 | 0.168 | 0.167 | 0.204 |
| **CD** |  | 0.000 | 0.159 | 0.202 |
| **EF** |  |  | 0.000 | 0.208 |
| **GH** |  |  |  | 0.000 |

**Table S3.** RMSD between the 4 different PSAT dimers present in the asymmetric unit in the OPS-soaked structure.

| **Dimer** | **AB** | **CD** | **EF** | **GH** |
| --- | --- | --- | --- | --- |
| **AB** | 0.000 | 0.196 | 0.193 | 0.231 |
| **CD** |  | 0.000 | 0.188 | 0.233 |
| **EF** |  |  | 0.000 | 0.234 |
| **GH** |  |  |  | 0.000 |

**Table S4.** RMSD between the 8 different PSAT chains present in the asymmetric unit in the substrate-free structure.

| **Chain** | **A** | **B** | **C** | **D** | **E** | **F** | **G** | **H** |
| --- | --- | --- | --- | --- | --- | --- | --- | --- |
| **A** | 0.000 | 0.160 | 0.167 | 0.153 | 0.165 | 0.165 | 0.177 | 0.204 |
| **B** |  | 0.000 | 0.156 | 0.162 | 0.166 | 0.172 | 0.169 | 0.209 |
| **C** |  |  | 0.000 | 0.157 | 0.167 | 0.165 | 0.188 | 0.203 |
| **D** |  |  |  | 0.000 | 0.142 | 0.155 | 0.175 | 0.204 |
| **E** |  |  |  |  | 0.000 | 0.151 | 0.186 | 0.210 |
| **F** |  |  |  |  |  | 0.000 | 0.183 | 0.188 |
| **G** |  |  |  |  |  |  | 0.000 | 0.204 |
| **H** |  |  |  |  |  |  |  | 0.000 |

**Table S5.** RMSD between the 8 different PSAT chains present in the asymmetric unit in the OPS-soaked structure.

| **chain** | **A** | **B** | **C** | **D** | **E** | **F** | **G** | **H** |
| --- | --- | --- | --- | --- | --- | --- | --- | --- |
| **A** | 0.000 | 0.194 | 0.189 | 0.188 | 0.183 | 0.190 | 0.206 | 0.223 |
| **B** |  | 0.000 | 0.197 | 0.194 | 0.189 | 0.196 | 0.185 | 0.227 |
| **C** |  |  | 0.000 | 0.192 | 0.214 | 0.177 | 0.228 | 0.240 |
| **D** |  |  |  | 0.000 | 0.175 | 0.174 | 0.211 | 0.229 |
| **E** |  |  |  |  | 0.000 | 0.172 | 0.217 | 0.232 |
| **F** |  |  |  |  |  | 0.000 | 0.208 | 0.218 |
| **G** |  |  |  |  |  |  | 0.000 | 0.208 |
| **H** |  |  |  |  |  |  |  | 0.000 |

**Table S6.** DALI structural comparison of the substrate-free PSAT against the PDB. The list of top 15 similar structures, sorted by Z-score, is shown. The list was generated by submitting a query in the online DALI server (<http://ekhidna2.biocenter.helsinki.fi/dali/>). The PSAT structure (PDB ID: 8a5v, chain A) was used as search model doing a PDB search request in the “all against all” mode.

| **#** | **PDB id-chain** | **z** | **rmsd** | **% id** | **Description** | **Organism** |
| --- | --- | --- | --- | --- | --- | --- |
| 1 | 3e77-A | 58.7 | 0.6 | 98 | PSAT | *Homo sapiens* |
| 2 | 6czx-A | 52.9 | 1.1 | 51 | PSAT 1  (chloroplastic) | *Arabidopsis thaliana* |
| 3 | 4xk1-A | 52.8 | 1.1 | 51 | PSAT | *Pseudomonas aeruginosa* |
| 4 | 7t7j-B | 52.6 | 1.2 | 48 | PSAT | *Klebsiella pneumoniae* |
| 5 | 1bjo-A | 52.4 | 1.2 | 48 | PSAT | *Escherichia coli* |
| 6 | 4azj-A | 52.1 | 1.2 | 45 | PSAT | *Alkalihalobacillus alcalophilus* |
| 7 | 3qbo-B | 51.7 | 1.3 | 49 | PSAT | *Yersinia pestis* |
| 8 | 1bt4-A | 51.2 | 1.3 | 42 | PSAT | *Niallia circulans* |
| 9 | 5f8v-A | 50.6 | 1.4 | 41 | PSAT | *Trichomonas vaginalis* |
| 10 | 3m5u-A | 50.2 | 1.4 | 37 | PSAT | *Campylobacter jejuni* |
| 11 | 6xdk-D | 50.2 | 1.4 | 43 | PSAT | *Stenotrophomonas maltophilia* |
| 12 | 5yb0-B | 47.8 | 1.6 | 39 | PSAT | *Entamoeba histolytica* |
| 13 | 3qm2-B | 45.6 | 1.4 | 48 | PSAT | *Salmonella enterica serovar Typhimurium* |
| 14 | 2fyf-A | 40.8 | 2.0 | 24 | PSAT | *Mycobacterium tuberculosis* |
| 15 | 3ffr-A | 40.7 | 1.8 | 22 | PSAT | *Cytophaga hutchinsonii* |

**Figure S8**: Amino acid sequence alignment of human PSAT from this work (8A5V) and from the previously deposited structure (3E77). The red arrow indicates the initial Met. The red box indicates the XGPY loop that is conserved among eukaryotes and prokaryotes^5^ (X indicates Ser/Ala/Pro, Y indicates Ser/Val/Ala). Numbering above the sequence begins with the start Met, the sequence used for cloning/tagging is not numbered.


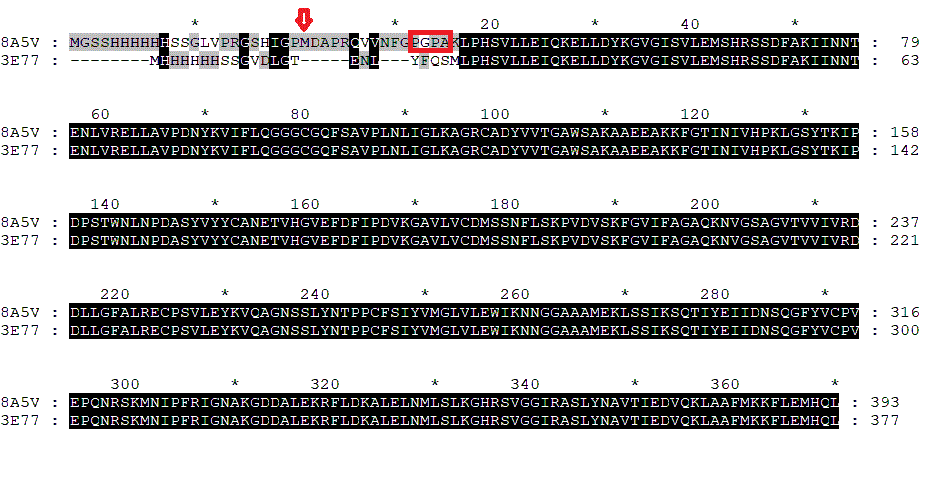


**Table S7 -** Protein-ligand interactions of the reaction intermediates. In the first row, the graphical models of three intermediates identified in the corresponding chains of OPS-soaked crystals are shown: ~~(~~PLP-K200 internal aldimine and substrate-free OPS (chain B); PLP-K200-OPS geminal diamine (chain E); PLP-OPS external aldimine (chain D). The residues involved in the interaction with either the cofactor (PLP) or the ligand (OPS) are listed in the left column. The interactions are grouped as hydrophobic, pi stacking, H-bonds and salt bridges. The background color indicates the functional group in the structure involved in the interaction.


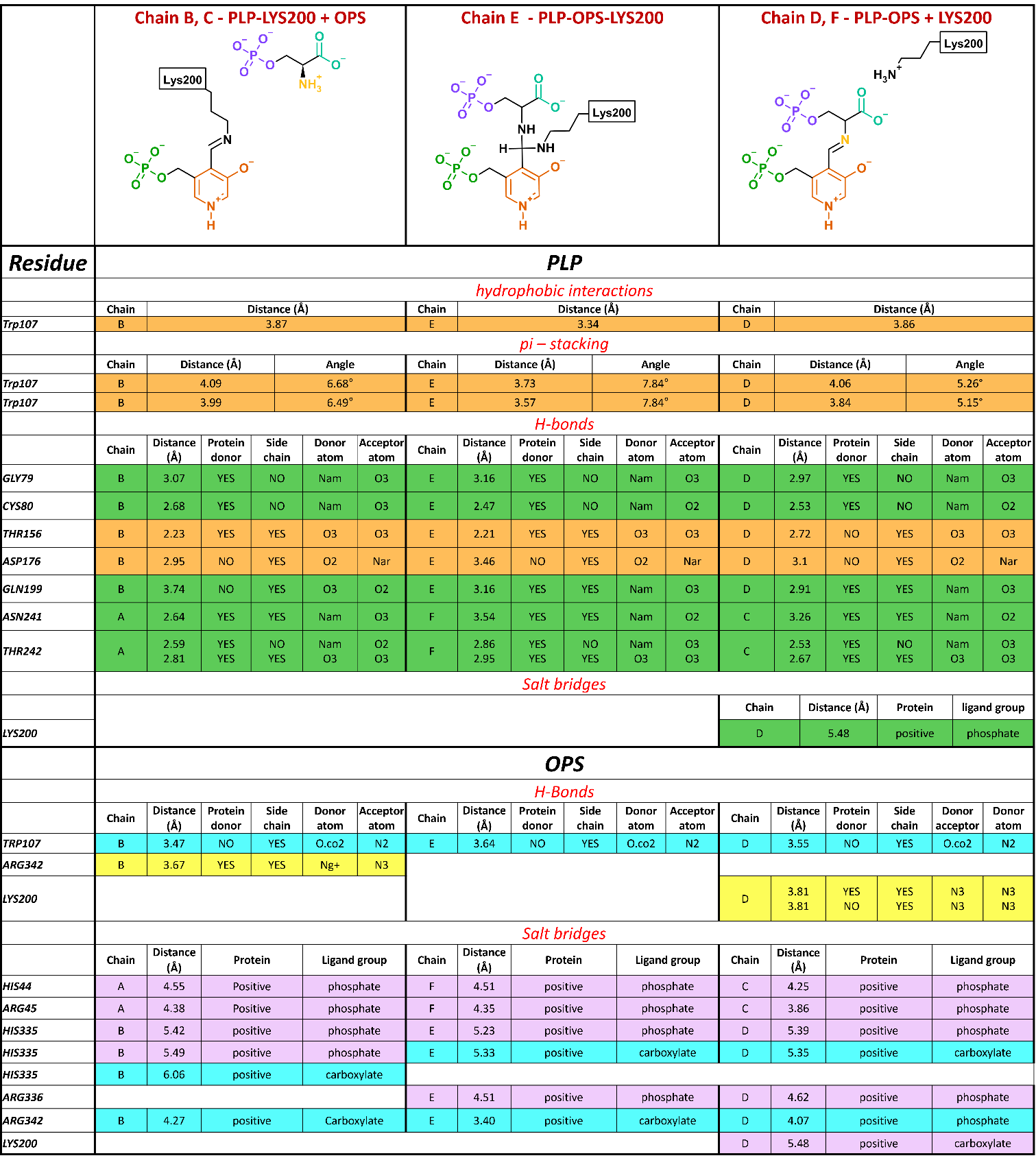


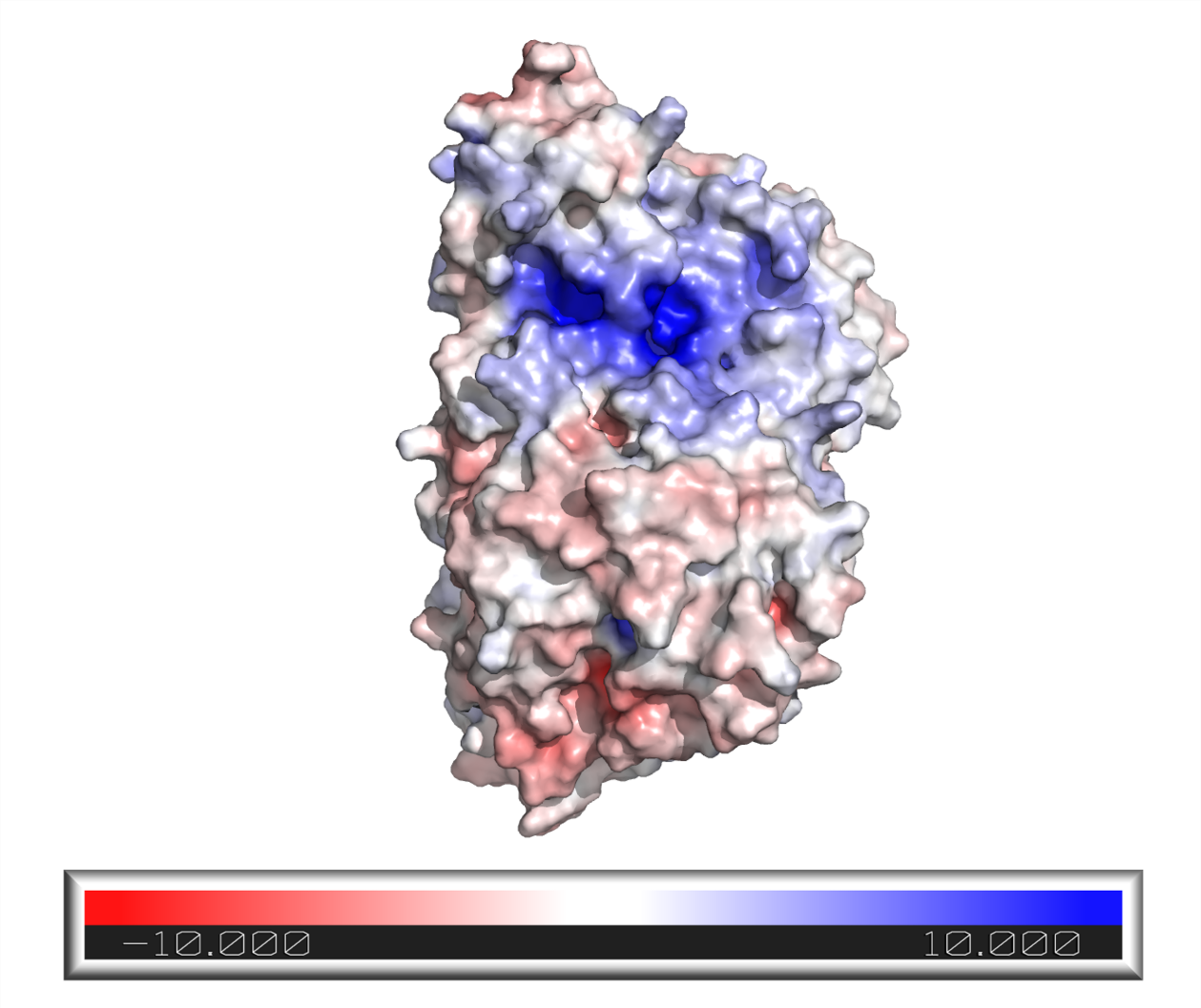


**Figure S9**: Molecular surface of human PSAT (PDB ID: 8A5V) colored according to electrostatics generated with the online server PDB2PQR and APBS^6^. Positive potentials are shown in blue and negative potentials in red (±10 kT/e).

**Table S8.** K_m_ values of PSAT from different species.

| **ORGANISM** | **K_m_ (mM)** | **Ref** |
| --- | --- | --- |
| ***B. taurus*** | 0.005 (3-PHP); 1.2 (L-Glu)  0.035 (OPS); 0.8 (α−KG) | ^7^ |
| ***E. histolytica*** | 0.037 (OPS); 0.043 (α−KG ) | ^8^ |
| ***O. aries*** | - 1. (L-Glu); 0.25 (3-PHP) | ^9^ |
| ***A. thaliana*** | 0.07 (L-Glu); 5 (3-PHP) | ^10^ |

.

**Table S9** - Data collection and refinement statistics. Values in parenthesis refer to the highest resolution shell.

|  | *PSAT* | *PSAT + O-PHOSPHOSERINE* |
| --- | --- | --- |
| **Data collection** | | |
| PDB ID | 8A5V | 8A5W |
| Space group | P2_1_ | P2_1_ |
| *Unit cell parameters* | | |
| a, b, c (Å) | 65.87, 206.27 135.54 | 65.43, 205.74, 135.75 |
| α, β, γ (°) | 90.00, 98.00, 90.00 | 90.00, 97.88, 90.00 |
| Number of molecules in the ASU | 8 | 8 |
| Cell volume (Å^3^) | 1823611.454 | 1810274.816 |
| Solvent content (%) | 56.75 | 59.10 |
| Matthews coefficient (Å^3^/Da) | 2.84 | 3.01 |
| Wavelength (Å) | 1.000 | 1.000 |
| Resolution (Å) | 81.78-2.46 | 134.47-2.78 |
|  | (2.78-2.46) | (3.11-2.78) |
| Number of unique reflections | 84262 (4214) | 60488 (3024) |
| R_merge_ | 0.150 (1.200) | 0.220 (1.282) |
| R_meas_ | 0.162 (1.300) | 0.238 (1.390) |
| R_pim_ | 0.062 (0.497) | 0.091 (0.533) |
| <I/σ(I)> | 12.5 (1.6) | 8.9 (1.5) |
| CC^1/2^ | 0.996 (0.599) | 0.993 (0.574) |
| Completeness - ellipsoidal (%) | 93.1 (65.7) | 93.2 (62.8) |
| Completeness - spherical (%) | 65.2 (10.8) | 67.6 (11.7) |
| Multiplicity | 6.8 (6.8) | 6.8 (6.7) |
|  | *PSAT* | *PSAT + O-PHOSPHOSERINE* |
| **Refinement** | | |
| Resolution (Å) | 48.14 -2.46 | 51.44-2.78 |
| Number of reflections | 84229 | 60462 |
| Number of reflections (R-Free) | 4117 | 2953 |
| R_work_/R_free_ (%) | 17.86 / 21.05 | 18.21 / 22.76 |
| *r.m.s. deviations* | | |
| bond length (Å) | 0.007 | 0.005 |
| bond angles (°) | 0.730 | 0.688 |
| *Ramachandran plot* | | |
| favored (%) | 97.33 | 96.61 |
| allowed (%) | 2.57 | 3.22 |
| outliers (%) | 0.10 | 0.17 |

**REFERENCES**

1. Basurko M-J, Marche M, Darriet M, Cassaigne A (1989) Catalytic properties and specificity of phosphoserine aminotransferase from beef liver. Biochem Soc Trans 17:787–788.

2. Lund K, Merrill DK, Guynn RW (1987) Purification and properties of phosphoserine aminotransferase from bovine liver. Arch Biochem Biophys 254:319–328.

3. Dubnovitsky AP, Kapetaniou EG, Papageorgiou AC (2005) Enzyme adaptation to alkaline pH: atomic resolution (1.08 A) structure of phosphoserine aminotransferase from Bacillus alcalophilus. Protein Sci 14:97–110.

4. Singh RK, Mazumder M, Sharma B, Gourinath S (2016) Structural investigation and inhibitory response of halide on phosphoserine aminotransferase from Trichomonas vaginalis. Biochim Biophys Acta 1860:1508–1518.

5. Coulibaly F, Lassalle E, Baker HM, Baker EN (2012) Structure of phosphoserine aminotransferase from Mycobacterium tuberculosis. Acta Crystallogr D Biol Crystallogr 68:553–563.

6. Jurrus E, Engel D, Star K, Monson K, Brandi J, Felberg LE, Brookes DH, Wilson L, Chen J, Liles K, et al. (2018) Improvements to the APBS biomolecular solvation software suite. Protein Science 27.

7. Basurko MJ, Marche M, Darriet M, Cassaigne A (1999) Phosphoserine aminotransferase, the second step-catalyzing enzyme for serine biosynthesis. IUBMB Life 48:525–529.

8. Ali V, Nozaki T (2006) Biochemical and functional characterization of phosphoserine aminotransferase from Entamoeba histolytica, which possesses both phosphorylated and non-phosphorylated serine metabolic pathways. Mol Biochem Parasitol 145:71–83.

9. Hirsch H, Greenberg DM (1967) Studies on phosphoserine aminotransferase of sheep brain. J Biol Chem 242:2283–2287.

10. Ho CL, Noji M, Saito M, Yamazaki M, Saito K (1998) Molecular characterization of plastidic phosphoserine aminotransferase in serine biosynthesis from Arabidopsis. Plant J 16:443–452.
